# Comparability of skeletal fibulae surfaces generated by different source scanning (dual‐energy CT scan vs. high resolution laser scanning) and 3D geometric morphometric validation

**DOI:** 10.1111/joa.13714

**Published:** 2022-06-25

**Authors:** Annalisa Pietrobelli, Rita Sorrentino, Veronica Notariale, Stefano Durante, Stefano Benazzi, Damiano Marchi, Maria Giovanna Belcastro

**Affiliations:** ^1^ Department of Biological, Geological and Environmental Sciences University of Bologna Bologna Italy; ^2^ Department of Cultural Heritage University of Bologna Ravenna Italy; ^3^ IRCCS Istituto Ortopedico Rizzoli Bologna Italy; ^4^ IRCCS Azienda Ospedaliero‐Universitaria di Bologna Policlinico S. Orsola Bologna Italy; ^5^ Department of Human Evolution Max Planck Institute for Evolutionary Anthropology Leipzig Germany; ^6^ Department of Biology University of Pisa Pisa Italy; ^7^ Centre for the Exploration of the Deep Human Journey University of the Witwatersrand Johannesburg South Africa

**Keywords:** 3D geometric morphometrics comparability, dual‐energy CT scans, high‐resolution surface scanning, human fibula

## Abstract

This work aims to test accuracy and comparability of 3D models of human skeletal fibulae generated by clinical CT and laser scanner virtual acquisitions. Mesh topology, segmentation and smoothing protocols were tested to assess variation among meshes generated with different scanning methods and procedures, and to evaluate meshes‐interchangeability in 3D geometric morphometric analysis. A sample of 13 left human fibulae were scanned separately with Revolution Discovery CT dual energy (0.625 mm resolution) and ARTEC Space Spider 3D structured light laser scanner (0.1 mm resolution). Different segmentation methods, including half‐maximum height (HMH) and MIA‐clustering protocols, were compared to their high‐resolution standard generated with laser‐scanner by calculating topological surface deviations. Different smoothing algorithms were also evaluated, such as Laplacian and Taubin smoothing. A total of 142 semilandmarks were used to capture the shape of both proximal and distal fibular epiphyses. After Generalized Procrustes superimposition, the Procrustes coordinates of the proximal and distal fibular epiphyses were used separately to assess variation due to scanning methods and the operator error. Smoothing algorithms at low iteration do not provide significant variation among reconstructions, but segmentation protocol may influence final mesh quality (0.09–0.24 mm). Mean deviation among CT‐generated meshes that were segmented with MIA‐clustering protocol, and laser scanner‐generated ones, is optimal (0.42 mm, ranging 0.35–0.56 mm). Principal component analysis reveals that homologous samples scanned with the two methods cluster together for both the proximal and distal fibular epiphyses. Similarly, Procrustes ANOVA reveals no shape differences between scanning methods and replicates, and only 1.38–1.43% of shape variation is due to scanning device. Topological similarities support the comparability of CT‐ and laser scanner‐generated meshes and validate its simultaneous use in shape analysis with potential clinical relevance. We precautionarily suggest that dedicated trials should be performed in each study when merging different data sources prior to analyses.

## Graphical abstract

Topological similarities support the comparability of CT‐ and laser scanner‐generated meshes of fibulae and validate its simultaneous use in shape analysis with potential clinical relevance.

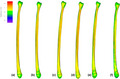

## 1 INTRODUCTION

Several digitizing techniques, such as laser scanning, digital three‐dimensional (3D) photogrammetry, computed tomography (CT) and micro‐CT scanning, are widely implemented in morphological analyses, to evaluate bone shape and metric features. Such approaches consist of effective, repeatable, non‐invasive diagnostic tools that allow the digitized object to be thoroughly examined without being physically manipulated, both in their external and internal geometry (Mantini & Ripani, 2009; Profico et al., 2019; Uldin, 2017; Weber & Bookstein, 2011; Weber et al., 2001; Weber, 2014, 2015).

Often, the digitized 3D surface meshes obtained by CT and laser scanner are interchangeably adopted together and merged in morphological analyses (e.g., Frelat et al., 2017; Mounier & Lahr, 2019; Sorrentino et al., 2021; Sorrentino, Carlson, et al., 2020). Several studies have compared surface meshes obtained through 3D surface scanning with meshes obtained through medical CT scanning (Adams et al., 2015; Brzobohatá et al., 2012; Choi et al., 2002; DeVries et al., 2008; Fahrni et al., 2017; Fourie et al., 2011; Lalone et al., 2015; Ramme et al., 2009; Waltenberger et al., 2021) or micro‐CT scanning (Teeter et al., 2012) finding consistency among methods in measurement accuracy. Indeed, Adams et al. (2015) attested 0.4 mm mean surface deviations among surface meshes of hominin fossils digitized with a laser scanner, clinical CT and micro‐CT scans. More recently, Waltenberger et al. (2021) compared surface meshes of human pelvises generated utilizing different digitizing methodologies (CT scans, laser scans, photogrammetry, MicroScribe digitizer) through a topological and landmark‐based geometric morphometric (GM) approach. In their work, the average deviation among the surface meshes spanned between 100–200 μm, and the GM results attested the comparability among 3D meshes acquired with CT scanning, 3D structured light scanning and photogrammetry (Waltenberger et al., 2021).

While most studies used the skull to investigate differences among scanning devices (e.g., Brzobohatá et al., 2012; Fahrni et al., 2017; Pinsky et al., 2006), others examined articular surfaces of long bones, revealing less than 0.4 mm average deviation among acquired elbow meshes (Lalone et al., 2015). Similar values were obtained when assessing mesh accuracy of the bones of the forearm (Oka et al., 2009). Considering the human femur, topological analyses on CT‐ and laser scanner‐generated meshes found a deviation spanning 0.71–0.79 mm (Gelaude et al., 2008; Soodmand et al., 2018; Stephen et al., 2021). Additionally, Stephen et al. (2021) analyzed also the tibia and found comparable deviation of CT vs. laser scanner generated meshes (less than 0.71 mm for tibiae).

Besides the scanning devices, other factors such as the choice of segmentation protocols may heavily influence the final outcome of mesh reconstructions, with potential impact on morphometric studies. Previous studies have tested the effect of bone segmentation procedures on mesh accuracy and comparability in morphometric analyses, including visual ‐based segmentation protocol, canny edge detection and other automated algorithms (Toro‐Ibacache, 2013; Gunz et al., 2012; Fourie et al., 2012; Engelbrecht et al., 2013; Rathnayaka et al., 2011; Ito, 2019). In particular, Ito (2019) found that single thresholding is often not suitable for a complex object with heterogeneous gray‐value distributions and had statistically significant effect on shape and size variation.

Furthermore, the choice of smoothing procedures and the number of the iterations used have also shown possible effect on capturing anatomical information of virtually acquired bones, with potential impact on morphometric analyses (Profico et al., 2016; Veneziano et al., 2018).

The present work will explore the impact of different scanning devices, segmentation protocols and smoothing procedures on the reconstruction of surface models to test their comparability in morphometric analyses, with the aim of providing new data on the comparison of digitizing methodologies of long bones. Specifically, we test the accuracy and comparability of CT‐ and laser scanner‐generated meshes in a sample of 13 modern human fibulae, considering the whole bone surface but also focusing on the extremities. The fibula has so far not been included in previous mesh accuracy assessments, despite its digitization in several biomechanical studies, alongside the tibia (e.g., Marchi, 2005, 2007, 2015b; Marchi, 2015a; Marchi et al., 2019, 2022). In addition, fibular 3D meshes are routinely utilized in reconstructive surgery, in the assessment of tibio‐fibular syndesmotic reductions performed after ankle fractures (e.g., Ebinger et al., 2013; Souleiman et al., 2021) and surgical planning of mandibular reconstructions from fibular free flaps (e.g., Damecourt et al., 2020; Ni et al., 2021; Ren et al., 2018).

We also aim to provide data on the whole virtual acquisition pipeline, by testing the impact of segmentation protocols (i.e., half‐maximum height and MIA‐clustering protocols), smoothing procedures (i.e., Laplacian and Taubin smoothing) and 3D landmark configuration repeatability in inter‐method comparisons. The results will add to our understanding of the use of 3D surface models obtained through different scanning techniques and procedures in 3D morphometric studies, with potential application in corrective surgical planning.

## 2 MATERIALS AND METHODS

### 2.1 The sample

The analyzed sample includes 13 left fibulae belonging to individuals from the Human Identified Skeletal Collection of Sassari, housed at the University of Bologna (Italy) and dating to the 19th–20th century. The fibulae were selected for their general good state of preservation, except for a case (SS23) intentionally selected for the presence of a small areas of cortical damage, to test the surface reconstruction performance in the case of a partially damaged bone. The human sample includes 8 males and 5 females spanning 20–45‐year‐old.

### 2.2 Data acquisition, segmentation protocols, and smoothing procedures

#### 2.2.1 Laser scanner acquisition

The sample of fibulae was scanned with ARTEC Space Spider 3D (Luxembourg), housed at the BONES Lab in the Department of Cultural Heritage of the University of Bologna. The ARTEC Space Spider 3D is a mobile and structured light laser scanner based on blue light technology, which uses trigonometric triangulation to calculate the distance among the points on the surface of the object and creates its relative point cloud. This machine provides a point‐accuracy of 0.05 mm and mesh resolution of 0.1 mm. Moreover, it acquires both geometry and texture data (1.3 megapixel, 24‐bits per pixel). The acquisition took place in two recording sessions for each specimen. The first scan was performed by holding the device approximately perpendicular to the surface of the fibular diaphysis, with one of the extremities that was in turn placed vertically into a polystyrene support with a cavity in the center that accommodates the epiphysis, and by rotating the turning table. The non‐supported epiphysis and part of the diaphysis were then acquired and subsequently a second scan was performed by inverting the fibular extremities. The integrated scanner software (Artec Studio 9) was utilized to merge the two sets of acquisitions, which were first cleaned and then manually aligned. These roughly aligned scans were then globally registered, with outlying points removed, and subsequently, a single polygonal 3D mesh is created with the Sharp Fusion algorithm, finally generating the 3D surface model that was then saved in .stl (Little Endian) format, following the procedures that are presented by the manufacturer user documentation.

#### 2.2.2 CT scan acquisition

All fibulae were also digitized through computed tomography (CT), utilizing a Revolution Discovery CT dual energy, with GSI Revolution and HD Revolution configurations, housed at Istituto Ortopedico Rizzoli (Bologna, Italy). The acquisition protocol chosen was Extremity GSI. Once the minimum Field of View (FOV) had been achieved to optimize the best resolution, an acquisition with polychromatic beam at 100 kV and 360 mA was performed with slice thickness and acquisition interval at 0.625 mm. A “Standard” reconstruction filter with WW 400 and WL 40 was selected. Then, two reconstructions with monochromatic beams were made. The first reconstruction is at 70 keV with “Detail” reconstruction filter, with superior detail of bone acquisition in respect to the “Bone” filter with WW 2000 and WL 350. The second reconstruction is at 40 keV, always using the “Detail” filter with WW 400 and WL 400. The obtained reconstructed DICOM (16‐bit grayscale, signed, voxel size ranging from 0.39 to 0.507 × 0.625) images at 40 keV, chosen for subsequent analyses, were then processed with Avizo 9.2 (Thermo Fisher Scientific) for image segmentation.

#### 2.2.3 Image segmentations

On the CT‐generated meshes, we applied the half‐maximum height (HMH) protocol outlined by Spoor et al. (1993), following the modified version detailed in Coleman and Colbert (2007), which included calculating the HMH for a row of pixels on the bone‐to‐air transition for 10 randomly selected slices, then averaged and applied to the whole stack. In addition, we also opted to visually segment the image stack utilizing a visual single‐threshold based technique including voxels above −550, −600, −650 and −700 grayscale value intensity thresholds, to evaluate how possible differences of the chosen segmentation procedure influenced the 3D reconstruction overall quality, compared to the same surface data obtained by laser scanner. For both HMH and visual single‐threshold based segmentations, thresholding was performed in Avizo 9.2 (Thermo Fisher Scientific, Waltham), determining grayscale intensity of selected voxels with either “histogram” or “magic wand” tools in the segmentation editor.

Moreover, we tested an additional segmentation protocol based on a machine‐learning approach, MIA‐clustering, which implements a clustering algorithm sorting the voxels of an image into clusters. This approach evaluates a global c‐means clustering, and then separates the image into overlapping regions where more c‐means iterations perform this sorting both globally and locally (Dunmore et al., 2018). For our sample, we tested different numbers of clusters (from 3 to 5) at a 2% threshold and selected for subsequent analysis the image segmented utilizing a grid size of 3 pixels and 4 clusters, which allowed us to better represent intensity inhomogeneity of the input CT‐ scans images.

Lastly, an isosurface (.stl in Little Endian format) was generated for each segmentation protocol.

#### 2.2.4 Smoothing procedures

Several smoothing procedures were tested on one specimen by calculating distances from the reconstructed CT‐generated mesh and post‐processed with different smoothing procedures to the homologous high‐resolution laser scanner‐generated mesh. Prior to smoothing testing, CT‐generated meshes were segmented with the same segmentation protocol (HMH), chosen arbitrarily to reduce possible variation induced by different segmentation protocols and to control only for smoothing differences. The smoothing procedures tested are Laplacian smoothing with or without surface preservation and Taubin smoothing (Taubin, 1995) with different number iterations (0.5; 1; 3; 5), generated in Meshlab 2020.12 (Cignoni et al., 2008). Distances from CT‐generated meshes smoothed with different procedures to the laser scanner‐generated mesh were calculated, prior to alignment, following the procedures detailed below for all meshes comparisons.

### 2.3 Surfaces comparisons

The comparison among 3D surface meshes was performed in R v. 4.0.3. (R Core Team, 2020). Both CT‐ and laser scanner‐generated meshes were uploaded and aligned by their Principal Axes with the function pcAlign() from the package “Morpho” (Schlager, 2017), using the CT‐generated mesh as target for the alignment of the laser scanner‐generated mesh, with an optimization procedure that minimizes root mean square errors (RMSE) between reference and target mesh, by testing all possible axes alignments with a rigid iterative closest point (ICP) procedure. Both iterations and subsampled points were set at 200, to compute the optimization procedure.

Topological analysis of distances among vertices of the CT‐ and laser scanner‐generated meshes was then implemented by the function meshDist() from the package “Morpho” (Schlager, 2017), with the aligned laser‐scanner generated mesh as the reference and the CT‐generated mesh as the target. Mesh vertices distances were then represented by a polychrome scale with values ranging from 2.50 to −2.50 mm. Distances up to 0.20 mm were depicted in green and considered not relevant. Descriptive statistics (mean, standard deviation, and maximum displacement of surface scan mesh surface from the CT mesh) of distance among the vertices of the triangles that form meshes, were also calculated. We also provide %variation among CT‐ and surface scanner‐generated meshes, calculated by dividing mean variation by maximum diameter at fibular midshaft multiplied by 100 (Table S1).

### 2.4 Geometric morphometric analysis

In addition to topological analysis, we assessed geometric distances between CT‐ and surface scanner‐generated meshes by adopting a geometric morphometric approach based on fixed landmarks and curves and surface semilandmarks, specifically designed for evaluating the accuracy of the two meshes at epiphyseal areas. Procrustes distances between landmarked surfaces generated by laser scanner and those generated by a CT‐scanner were analysed. For each specimen, the laser scanner‐generated meshes were compared to CT‐scan generated meshes, which were segmented by MIA‐clustering and HMH protocols separately. Additionally, a second digitization of the surface scanner‐generated mesh was included in this analysis, to quantify possible intra‐observer error due to template repetability and not to due to scanning device.

**FIGURE 1:**
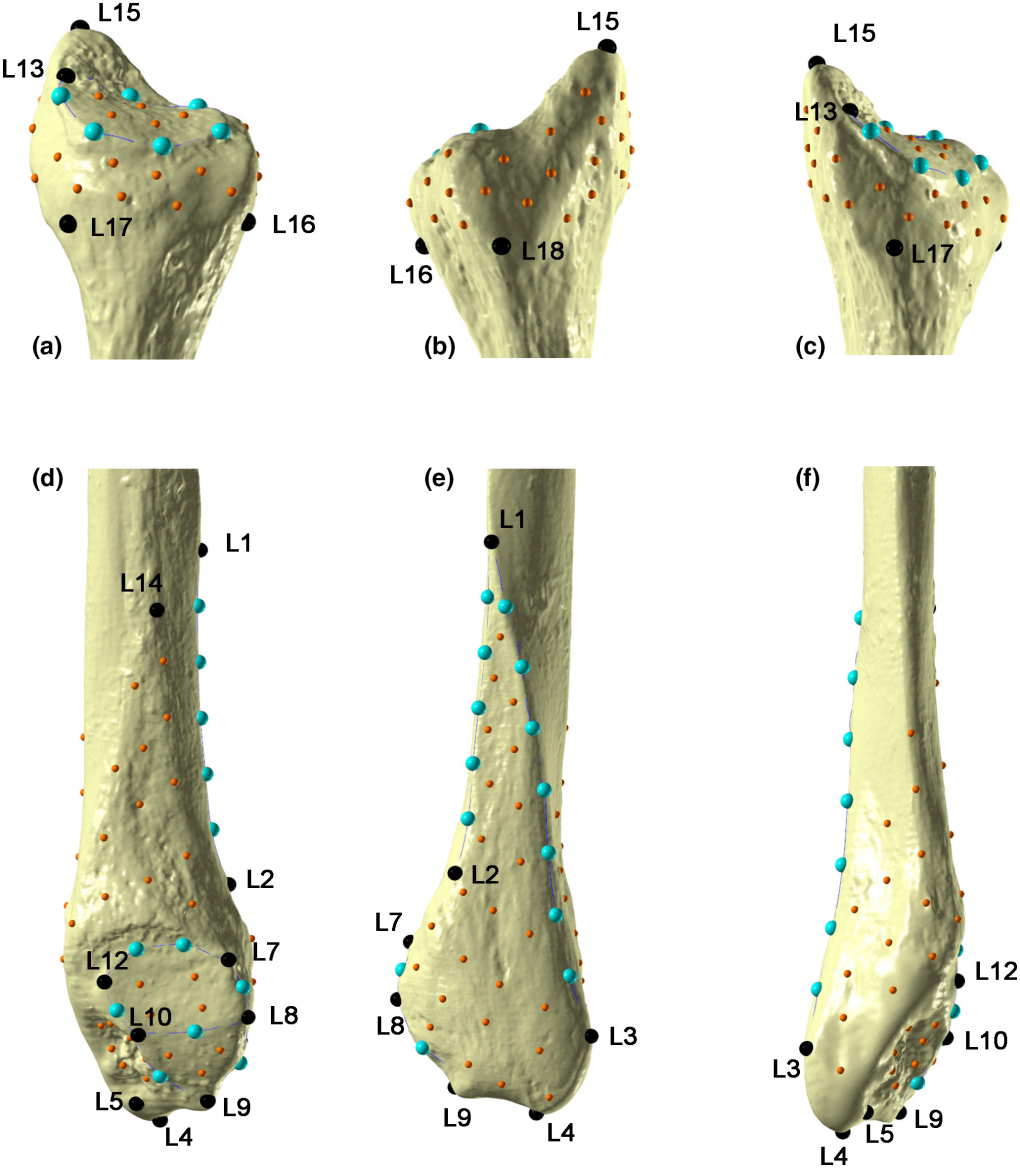
Landmarks (black), curve (light blue) and surface semilandmarks (orange) digitized on a left fibula (a, medial view, proximal; b, lateral view, proximal; c, postero‐medial view, proximal; d, medial view, distal; e, lateral view, distal; f, posterior view, distal). See Table 1 for a detailed description of the anatomical landmarks.

A template of 142 landmarks and semilandmarks (Table 1 and Figure 1), modified from a previous version published by Marchi et al. (2022), was applied to targets (both CT‐ and laser scanner‐generated models) utilizing Viewbox 4 software (dHal Software). Curve and surface semilandmarks were allowed to slide along the curves/surfaces to minimize the thin‐plate spline (TPS) bending energy between the target and the template obtaining geometrically homologous semilandmarks (Slice, 2005; Gunz & Mitteroecker, 2013). The set of semilandmarks for the proximal epiphysis (*N* = 94) and for the distal epiphysis (*N* = 48) were separated in order to perform two separate Generalized Procrustes Analysis (GPA) using the R package “geomorph” version 3.0.7 (Adams et al., 2018). As a result, the semilandmark coordinates were superimposed with scale, position and orientation standardized, with semilandmarks being allowed to slide with each recursive update of the Procrustes consensus (Gunz et al., 2005; Mitteroecker & Gunz, 2009; Rohlf & Slice, 1990; Slice, 2005; Sorrentino et al., 2020,b).

**TABLE 1:** Fibular landmarks and (semi)landmarks identification, definition, and number

Landmarks	Definition	
L1	Point where the *fibular anterolateral border* divides into two ridges: the proximal apex of the *subcutaneous triangular surface* (STS)	
L2	Most medial point of the medial border of the STS	
L3	Most lateral point of the lateral border of the STS	
L4	Most distal point of the *lateral malleolus* in anterior view	
L5	Most distal point of the posterior border of the *malleolar fossa*	
L7	Most anterior point on the anterior border of the *proximal fibular‐talar articular facet* (PAF)	
L8	Point between the anterior border of PAF and the *anterior border of distal fibular‐talar articular facet* (DAF)	
L9	Most distal point of DAF	
L10	Most proximal point on the posterior border of DAF	
L12	Most posterior point of the proximal border of PAF	
L13	Most proximal point of *proximal tibio‐fibular articular facet*	
L14	Most proximal point of *interosseous tibio‐fibular ligament* (ILA*)* insertion	
L15	Most proximal point on styloid process of fibular head in medial view	
L16	Most antero‐proximal point on anterior border in medial view (above fibular neck)	
L17	Most postero‐proximal point on posteromedial border in medial view (above fibular neck)	
L18	Most postero‐proximal point on posterior border in lateral view (above fibular neck)	

Curves	Definition	Number of semi‐landmarks
C_1‐ > 2	Medial border of the *subcutaneous triangular surface* (STS)	5
C_1‐ > 3	Posterior border of the STS	7
C_7‐ > 8	Anterior border of the *proximal fibular‐talar articular facet* (PAF)	1
C_8‐ > 9	Anterior border of the *distal fibular‐talar articular facet* (DAF)	1
C_9‐ > 10	Posterior border of the DAF	1
C_10‐ > 12	Posterior border of the PAF	1
C_8‐ > 10	Border between the PAF and DAF	1
C_13‐ > 13	Outline of *proximal tibio‐fibular articular facet*	6
C_7‐ > 12	Proximal border of the PAF	2

Surfaces	Definition	Number of semi‐landmarks
SSML_malleolar fossa	Surface of the *malleolar fossa*, attachment site of the *transverse tibiofibular* and *posterior talofibular ligaments*.	7
SSML_ILA	Attachment surface of *interosseous tibio‐fibular ligament* and part of *interosseous membrane* (ILA)	13
SSML_fibular groove	Groove for tendons of m. *peroneus longus* and m. *tertius* and attachment site of *posterior tibiofibular ligament*.	13
SSML_STS	*Subcutaneous triangular surface* (STS)	24
SSML_FiTal1Ar	Proximal *fibular‐talar articular facet* (PAF)	4
SSML_FiTal2Ar	Distal *fibular‐talar articular facet* (DAF)	3
SSML_head	Proximal *tibio‐fibular articular surface*	5
SSML_prox_ep	Surface of *proximal epiphysis*	32

Procrustes coordinates for each fibular epiphysis were then analyzed by Principal Component Analysis (PCA) to explore shape variations among specimens, differentiated by acquisition methodology, including for each individual the CT‐generated meshes segmented with both HMH and MIA‐clustering protocols, the surface scanner‐generated mesh and a second landmark digitalization replica of the latter. Procrustes ANOVA was then adopted to test shape differences among scanning devices, utilizing Procrustes distances among specimens digitized with different scanning source, with a residual randomization procedure (RRPP = T, iterations = 1′000), with the R package “geomorph” version 3.0.7 (Adams et al., 2018). Initially, we performed a Procrustes ANOVA on shape distances among all specimens and its interaction with relative scanning devices in the model formula (shape ~ device), considering CT‐generated meshes segmented with HMH and MIA‐clustering protocols as a group and both laser scanner replicates as another group (i.e., CT vs. LASER). Additionally, to exclude the influence of intra‐observer error on shape distances due to template repeatability and not solely to surface acquisition and segmentation method, a second Procrustes ANOVA (shape ~ method) was computed, this time separating CT‐generated meshes segmented with HMH and MIA‐clustering protocols and laser scanner replicates separately (i.e., CT‐HMH vs. CT‐MIA vs. LASER vs. LASER REPLICA).

Furthermore, a Post‐hoc test was calculated for the latter Procrustes ANOVA, to evaluate the interaction among CT‐generated meshes segmented with HMH and MIA‐clustering protocols and laser scanner replicates, with pairwise function from the RRPP package (Collyer et al., 2015; Collyer & Adams, 2018). This procedure controls for Type I errors in performing multiple tests, as the RRPP method performs the exact same random placement of residuals for every test statistic calculated, allowing us to consider these pairwise comparisons as separate inferences from the same test (Collyer et al., 2015). The replicate design allowed us to assess whether variation due to scanning methods could be proxy of operator error (i.e., intra‐observer error), therefore denouncing similar (possibly negligible) approximation. Finally, size comparisons among CT‐generated meshes segmented with HMH and MIA‐clustering protocols and the two laser scanner replicates were calculated using the centroid size, which is the square root of the summed squared distances between each semilandmark and the centroid of the semilandmark configuration (Slice, 2005). Centroid size distributions were evaluated visually by box‐plots and differences among CT‐generated meshes segmented with HMH and MIA‐clustering protocols and laser scanner replicates were quantified by one‐way ANOVA.

Landmarks and semilandmarks coordinates and sample list used to describe the specimens of the study are available (DOI: 10.5281/zenodo.6425379).

## 3 RESULTS

### 3.1 Smoothing procedures

Consistency among smoothing procedures was revealed, but better results were offered when Laplacian smoothing with surface preserve was applied (Table 2; Figure S1). Therefore, for further analysis we opted to follow the application of a Laplacian smoothing with surface preserve at 0.5 iteration, minimizing post‐processing procedures, to all CT‐generated meshes.

**TABLE 2:** Descriptive statistics (mean. Standard deviation. Minimum and maximum values) of distances among vertexes of CT‐generated mesh of SS163, segmented with HMH protocol, and subjected to different smoothing protocols, and its respective laser scanner‐ generated mesh. Smoothing protocols evaluated presence/absence of surface preserve filter and a different number of iterations (0.5, 1, 3, 5)

	Mean (mm)	Sd (mm)	Max (mm)	Min (mm)
Laplacian smoothing with SP				
0.5	0.51	0.27	1.71	0
1	0.51	0.28	1.79	0
3	0.51	0.29	1.75	0
5	0.51	0.32	1.76	0
Range	0.00	0.05	0.08	0.00
Laplacian smoothing				
0.5	0.52	0.29	1.73	0
1	0.52	0.31	1.97	0
3	0.55	0.25	1.97	0
5	0.52	0.27	2.1	0
Range	0.03	0.06	0.37	0.00
Taubin smoothing				
0.5	0.51	0.28	1.80	0
1	0.52	0.28	1.77	0
3	0.53	0.28	1.78	0
5	0.53	0.31	1.94	0
Range	0.02	0.03	0.17	0
No smoothing	0.51	0.27	1.71	0

Abbreviation: SP, surface preserve.

### 3.2 Topological and visual meshes comparison

**FIGURE 2:**
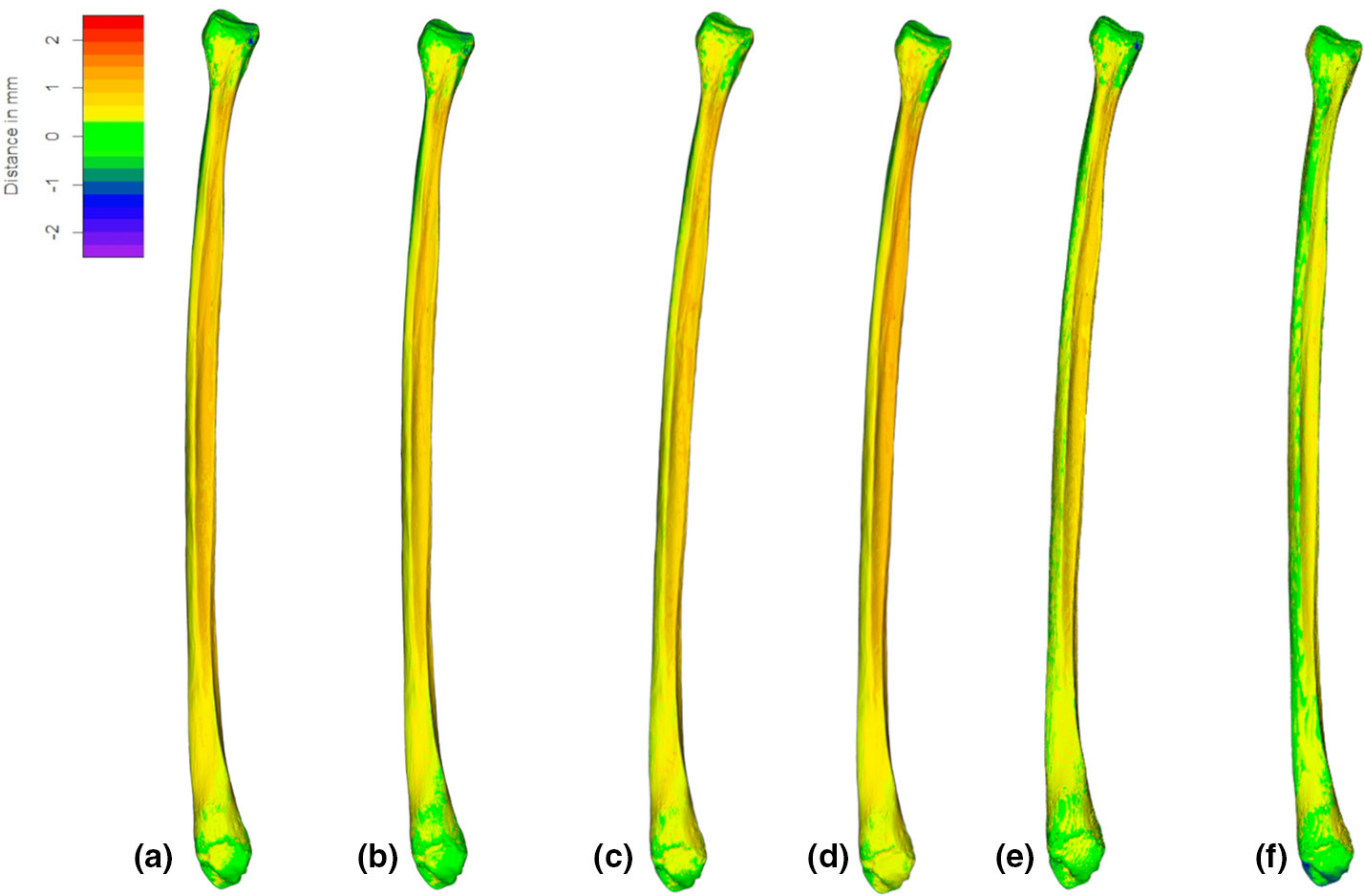
Computed tomography (CT) ‐generated meshes for every segmentation protocol for a representative specimen (SS163), with relative distances (−2.5 to 2.5 mm, from violet to red; in white, areas where deviations exceed this range), from their laser‐scanner generated equivalent. (a), CT segmented at −550 grayscale intensity; (b), CT segmented at −600 grayscale intensity; (c), CT segmented at −650 grayscale intensity; (d), CT segmented at −700 grayscale intensity; and e, CT segmented with half maximum height protocol (HMH) and f, MIA‐clustering protocol (MIA). Other specimens are represented in Figures S2–S14 in supplementary information.

Descriptive statistics for topological distances among every CT‐generated meshes segmented at different grayscale threshold values and with HMH and MIA‐clustering protocols, and laser scanner‐generated meshes are presented in Table 3. Topological differences among the CT‐generated meshes with different segmentations and the laser scanner‐generated meshes are presented in Figure 2. Comprehensively, the mean deviation between CT‐generated meshes and laser scanner‐generated meshes, considering all individuals at different segmentation protocols, vary between 0.35 mm and 0.70 mm, while maximum deviation values vary between 1.23–4.16 mm (Table 3).

**TABLE 3:** Descriptive statistics (mean, standard deviation, and maximum values) of distances calculated among CT‐ and laser scanner‐ generated meshes (vertexes) for the 13 specimens analysed. %var is calculated as (mean distance/max diameter at midhsft*100). Maximum midshaft diameters at midshaft for each specimen are presented in Table S1. For each specimen, segmentations at four different grayscale values are considered (−550, −600, −650, and −700; for SS163: −500, −550, −600, −650). For each specimen, total individual range variation in deviations considering all segmentation protocols is indicated at the bottom of each specimen's section. The total average of deviations considering every segmentation with half maximum height (HMH) and MIA‐clustering protocols for all specimen, are indicated in the last rows. Values are in mm

	Mean (mm)	Sd (mm)	Max (mm)	%		Mean (mm)	Sd (mm)	Max (mm)	%
*SS4*					*SS95*				
**−550**	0.59	0.25	1.39	3.44	**−550**	0.48	0.32	3.33	3.45
**−600**	0.62	0.25	1.49	3.61	**−600**	0.52	0.33	3.52	3.74
**−650**	0.68	0.26	1.51	3.96	**−650**	0.56	0.37	3.81	4.03
**−700**	0.68	0.26	1.52	3.96	**−700**	0.6	0.38	3.7	4.32
**MIA**	0.56	0.2	1.57	3.26	**MIA**	0.4	0.31	2.99	2.88
**HMH**	0.56	0.22	1.5	3.26	**HMH**	0.46	0.31	3.04	3.31
**range**	0.12	0.06	0.18	0.7	**range**	0.2	0.07	0.82	1.41
*SS14*					*SS105*				
**−550**	0.48	0.3	4.06	3.38	**−550**	0.63	0.27	1.83	3.57
**−600**	0.51	0.27	3.3	3.59	**−600**	0.64	0.26	1.72	3.63
**−650**	0.54	0.29	2.57	3.81	**−650**	0.68	0.28	1.8	3.85
**−700**	0.59	0.29	2.46	4.16	**−700**	0.7	0.27	1.88	3.97
**MIA**	0.39	0.24	1.94	2.75	**MIA**	0.51	0.33	2.18	2.89
**HMH**	0.46	0.32	4.16	3.24	**HMH**	0.61	0.28	1.75	3.46
**range**	0.2	0.08	2.22	1.41	**range**	0.19	0.07	0.46	1.08
*SS23*					*SS115*				
**−550**	0.54	0.55	3.76	3.3	**−550**	0.55	0.2	1.23	3.44
**−600**	0.5	0.42	3.34	3.06	**−600**	0.58	0.21	1.33	3.63
**−650**	0.5	0.38	3.03	3.06	**−650**	0.6	0.2	1.25	3.75
**−700**	0.5	0.36	3.6	3.06	**−700**	0.63	0.2	1.55	3.94
**MIA**	0.45	0.29	2.26	2.75	**MIA**	0.4	0.16	1.27	2.5
**HMH**	0.47	0.41	3.62	2.87	**HMH**	0.54	0.22	1.39	3.38
**range**	0.09	0.26	1.5	0.55	**range**	0.23	0.06	0.32	1.62
*SS26*					*SS163*				
**−550**	0.59	0.27	2.04	3.5	**−550**	0.53	0.31	1.7	3.7
**−600**	0.61	0.27	1.88	3.61	**−600**	0.54	0.31	1.54	3.77
**−650**	0.64	0.26	2.01	3.79	**−650**	0.58	0.27	1.48	4.05
**−700**	0.66	0.27	2.01	3.91	**−700**	0.66	0.36	1.73	4.61
**MIA**	0.56	0.26	2.03	3.32	**MIA**	0.42	0.21	1.48	2.94
**HMH**	0.58	0.26	2.04	3.44	**HMH**	0.51	0.27	1.71	3.56
**range**	0.10	0.01	0.16	0.59	**range**	0.24	0.10	0.23	1.68
*SS28*					*SS215*				
**−550**	0.46	0.28	3.03	4.14	**−550**	0.58	0.22	1.66	4.19
**−600**	0.49	0.25	2.19	4.41	**−600**	0.61	0.24	1.92	4.41
**−650**	0.47	0.25	2.24	4.23	**−650**	0.63	0.27	2.11	4.56
**−700**	0.54	0.27	1.49	4.86	**−700**	0.67	0.33	2.9	4.84
**MIA**	0.39	0.23	1.63	3.51	**MIA**	0.45	0.22	1.86	3.25
**HMH**	0.45	0.26	2.93	4.05	**HMH**	0.57	0.26	2	4.12
**range**	0.15	0.05	1.54	1.35	**range**	0.22	0.11	1.24	1.55
*SS48*					*SS224*				
**−550**	0.44	0.22	3.32	3.06	**−550**	0.45	0.26	2.44	2.86
**−600**	0.45	0.2	2.99	3.13	**−600**	0.49	0.28	2.86	3.12
**−650**	0.48	0.2	2.75	3.34	**−650**	0.53	0.28	2.79	3.37
**−700**	0.54	0.23	2.37	3.76	**−700**	0.58	0.28	2.71	3.69
**MIA**	0.36	0.17	2.12	2.5	**MIA**	0.41	0.24	1.99	2.61
**HMH**	0.41	0.23	3.39	2.85	**HMH**	0.46	0.24	2.16	2.92
**range**	0.18	0.06	1.27	1.25	**range**	0.17	0.04	0.87	1.08
**Average MIA**	0.42	0.22	1.78	2.8	*SS234*				
**Average HMH**	0.45	0.27	2.29	3.02	**−550**	0.37	0.27	3.81	2.55
					**−600**	0.38	0.24	3.29	2.62
					**−650**	0.42	0.25	2.79	2.89
					**−700**	0.46	0.27	1.47	3.17
					**MIA**	0.35	0.21	1.27	2.41
					**HMH**	0.36	0.53	2.54	2.48
					**range**	0.11	0.32	2.54	0.76

For each individual, some degree of variation is seen among all its CT‐based meshes generated from different segmentations. Individually, the range of variation of the deviation between CT‐generated meshes and surface scanner‐generated meshes measured for different segmentation protocols was between 0.09–0.24 mm, and for maximum deviation valueswas between 0.16–2.54 mm (Table 3). While regions of variations (i.e., location on bone surface) within each individual accounted by different segmentation protocols are quite subtle, both CT‐generated meshes segmented with HMH and MIA‐clustering protocols seem to better represent a larger portion of the diaphyses and epiphyseal areas, compared to other segmentation (−550, −600, −650 and −700 grayscale value intensity thresholds) (Figure 2, Figures S2–S12).

**FIGURE 3:**
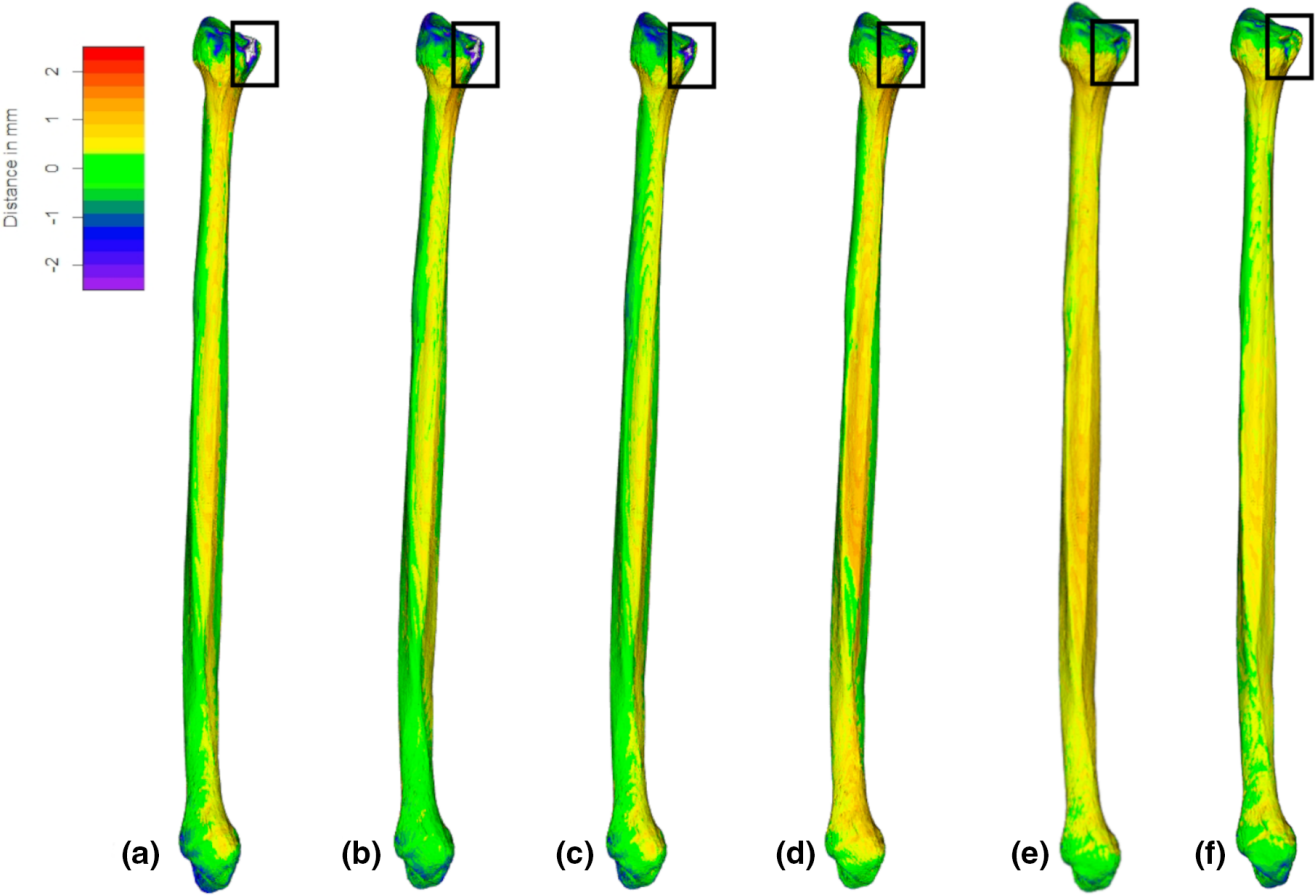
Computed tomography‐generated meshes on one specimen (SS23, left fibula) segmented with every segmentation protocol with relative distances (−2.5 to 2.5 mm, from violet to red; in white, deviations exceed this range), from its laser‐scanner generated equivalent; (a) CT segmented at −550 grayscale intensity; (b) CT segmented at −600 grayscale intensity; (c) CT segmented at −650 grayscale intensity; (d) CT segmented at −700 grayscale intensity; and (e) CT segmented with half maximum height protocol (HMH) and (f) MIA‐clustering protocol (MIA). Note the small area of cortical defect (black square).

For specimen SS23 (Figure 3), taken singularly as an example due to the presence of a small cortical defect, we observe a more evident difference in regional distribution of variations accounted by segmentation protocol, located on the antero‐medial portion of the fibular head, the styloid process, the proximal tibio‐fibular articular facet, the distal half of the interosseous border and the medial surface, the area of attachment of the interosseous ligament and the malleolar fossa. The small area of cortical loss at proximal epiphysis exceeded the range of mesh deviations in the first two segmentations and is thus mostly colored in white (Figure 3a–c), but is captured by the last three protocols (Figure 3d–f).

Indeed, for all specimens the CT‐generated mesh is optimized when the MIA‐clustering segmentation protocol is adopted, followed by HMH (Table 2). Deviation between CT‐generated meshes segmented with MIA‐clustering protocol and laser scanner‐generated ones ranges between 0.35 and 0.56 mm of mean, while maximum deviation values are within a range of 1.27 and 2.99 mm. Overall, the average of the mean deviation among CT‐generated meshes that were segmented with MIA‐clustering protocol and laser scanner‐generated ones is 0.42 mm, while the average of maximum deviations is 1.78 mm.

**FIGURE 4:**
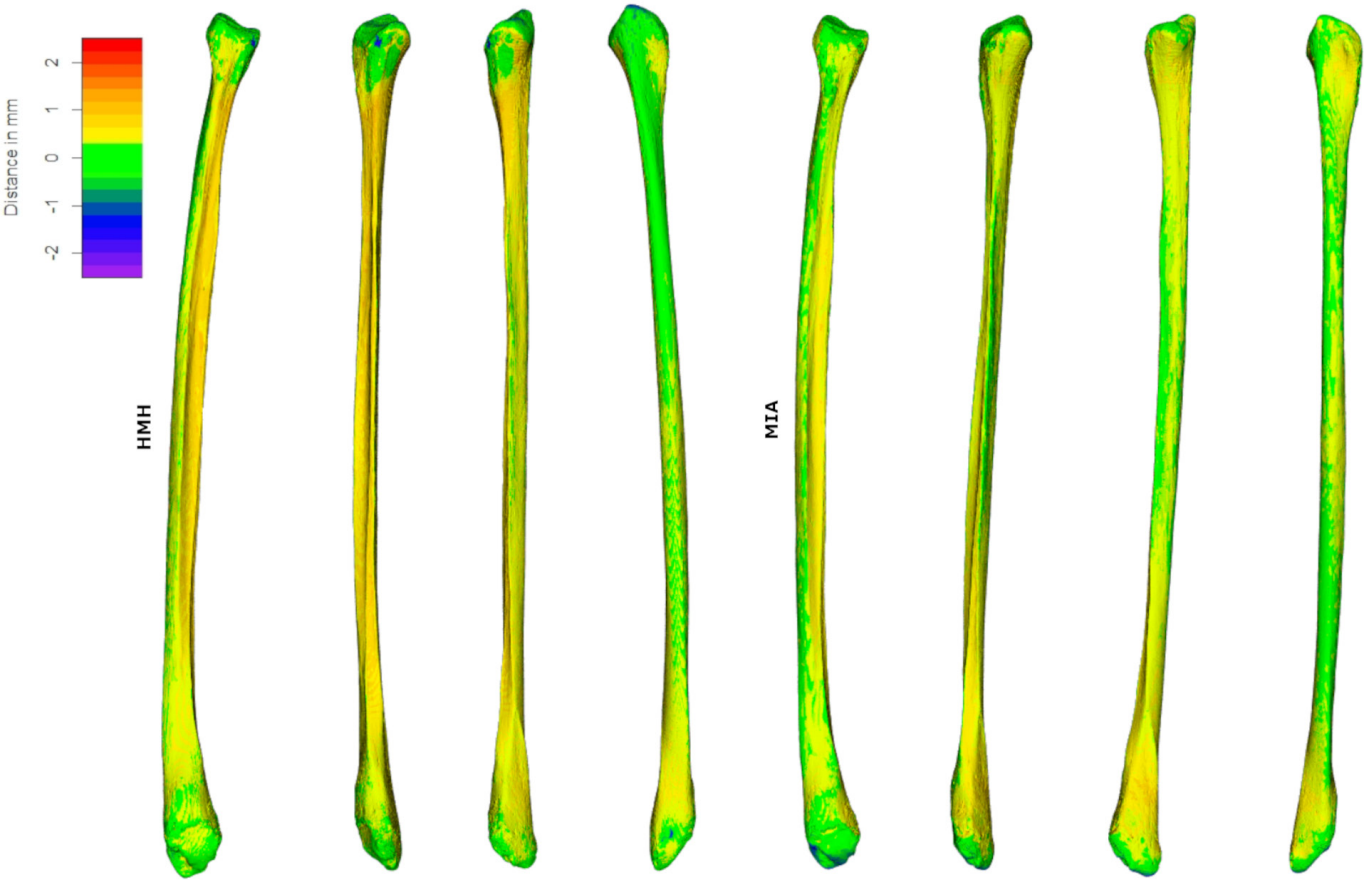
Computed tomography‐generated meshes on one representative specimen (SS163, left fibula) segmented with half maximum height protocol (HMH) and f, MIA‐clustering protocol (MIA), with relative distances (−2.5 to 2.5 mm, from violet to red; in white, deviations exceed this range), from its laser‐scanner generated equivalent, seen in medial, anterior lateral, and posterior view (from left to right).

Indeed, taking as reference specimen SS163, considered representative of the variations seen in all specimens (Figure 4) for both HMH and MIA‐clustering protocols there are areas with displacements from 0.2 to −0.2 mm, visible in green, in most of the posterior surface fibular diaphysis, while anterior and medial surfaces are slightly more displaced than the former, staying however within maximum 0.50 mm distance. The lowest divergence between CT and laser scanners is observed at the level of epiphyses, at the proximal tibio‐fibular articular facet and the distal fibular‐talar articular facet, whereas some degree of displacement is found around the fibular head, and at the superior border of the proximal fibular‐talar articular facet.

### 3.3 3D GM analysis for meshes comparison and intra‐observer error

**FIGURE 5:**
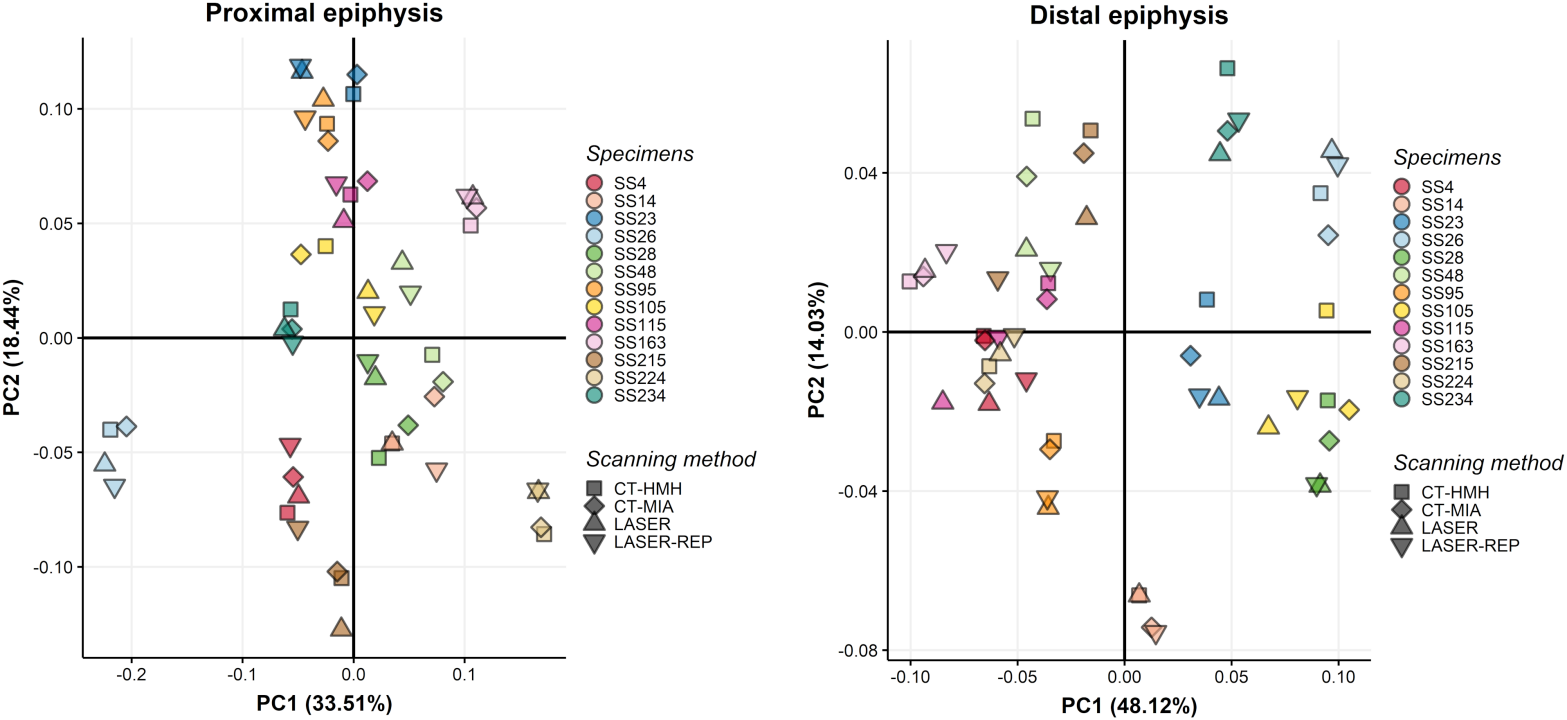
Principal component analysis plot considering landmark configurations applied to computed tomography (CT)‐generated segmented with HMH (square), and with MIA‐clustering (rhombus), and laser scanner‐generated individuals and replicates (triangles) for the distal and the proximal epiphysis. PC1: Principal component 1; PC2: Principal component 2.

The PCA plots of the shape coordinates for the proximal and distal epiphysis show that the CT‐generated specimens segmented with both HMH and MIA‐clustering protocols either overlapped or clustered in the very proximity of the homologous individual digitized with laser scanner and its replica (Figure 5; Figures S13 and S14). Results are corroborated by the Procrustes ANOVA for both distal and proximal epiphysis (Table 4) comparing shape variation due to scanning device considering CT‐generated meshes (pooled meshes segmented with HMH and MIA protocols) and laser scanner (original and replicates combined together), with no significant differences found. Considering both proximal and distal epiphyses, only 1.38%–1.43% of variance (*Rsq*) is explained by differences in scanning device, while 98.57%–98.62% of variance is explained by residuals, suggesting that biological variations may account for the large proportion of this variance, independently from the scanning devices.

**TABLE 4:** Results of Procrustes ANOVA for distal and proximal epiphyses, considering computed tomography (CT)‐generated meshes segmented with HMH and MIA‐clustering protocols together and both laser scanner replicates together, comparing shape variation due to scanning device (i.e., CT vs. LASER)

Procrustes ANOVA	Df	SS	MS	Rsq	*F*	*p* value
Distal epiphysis						
Device	1	0.00605	0.0060516	0.01378	0.6986	ns
Residuals	50	0.43313	0.0086626	0.98622		
Total	51	0.43918				
Proximal epiphysis						
Device	1	0.01786	0.017857	0.0143	0.7253	ns
Residuals	50	1.23096	0.024619	0.9857		
Total	51	1.24881				

Moreover, the PCA plots for the proximal and distal epiphysis show that the replicates (digitized on the laser scanner meshes) maintain similar variances and plot closely to their homologous meshes coming from the first digitization on laser scanner (Figure 5). Indeed, when Procrustes ANOVA is computed considering CT‐generated meshes segmented with HMH and MIA‐clustering protocols and laser scanner replicates separately (Table 5), again no significant difference among all configurations emerged (*p* = >0.05 for both epiphyses). The Procrustes distance between LASER vs. LASER REPLICA is actually lower than distances among all other comparisons (Table 6), ultimately denoting low intra‐observer error in the application of the template. This result likely indicates that (1) the intra‐observer error is negligible, (2) and that the inter‐method comparisons are not influenced by repeating the template on the same individual (intra‐observer error).

**TABLE 5:** Results of Procrustes ANOVA for distal and proximal epiphyses, considering computed tomography (CT)‐generated meshes segmented with HMH and MIA‐clustering protocols and laser scanner replicates separately (i.e., CT‐HMH vs. CT‐MIA vs. LASER vs. LASER REPLICA)

Procrustes ANOVA	Df	SS	MS	Rsq	*F*	*p* value
Distal epiphysis						
Method	3	0.00807	0.0026917	0.01839	0.2997	ns
Residuals	48	0.43111	0.0089814	0.98161		
Total	51	0.43918				
Proximal epiphysis						
Method	3	0.02117	0.0070581	0.01696	0.276	ns
Residuals	48	1.22764	0.0255758	0.98304		
Total	51	1.24881				

**TABLE 6:** Post‐hoc Procrustes distances pairwise comparisons among computed tomography (CT)‐generated meshes segmented with HMH and MIA‐clustering protocols and two sets of laser scanner replicates considered separately. D = Procrustes distance; UCL = upper confidence limit

	*d*	UCL (95%)	*Z*	*p* value	Global *p* value
Distal epiphysis					
CT‐HMH: CT‐MIA	0.016164350	0.05585739	−2.608923	ns	ns
CT‐HMH: Laser1	0.024059392	0.05637040	−1.038789	ns	
CT‐HMH: Laser2	0.024081671	0.05640215	−1.042411	ns	
CT‐MIA: Laser1	0.022243496	0.05902505	−1.353978	ns	
CT‐MIA: Laser2	0.022798490	0.05591536	−1.278960	ns	
Laser1: Laser2	0.007071018	0.05844946	−6.105541	ns	
Proximal epiphysis					
CT‐HMH: CT‐MIA	0.01782615	0.08976858	−4.264950	ns	ns
CT‐HMH: Laser1	0.03426822	0.08563265	−1.721336	ns	
CT‐HMH: Laser2	0.03956952	0.08771015	−1.360360	ns	
CT‐MIA: Laser1	0.03783650	0.08875301	−1.517139	ns	
CT‐MIA: Laser2	0.04281501	0.08964866	−1.073916	ns	
Laser1: Laser2	0.01387727	0.08989073	−4.294620	ns	

**FIGURE 6:**
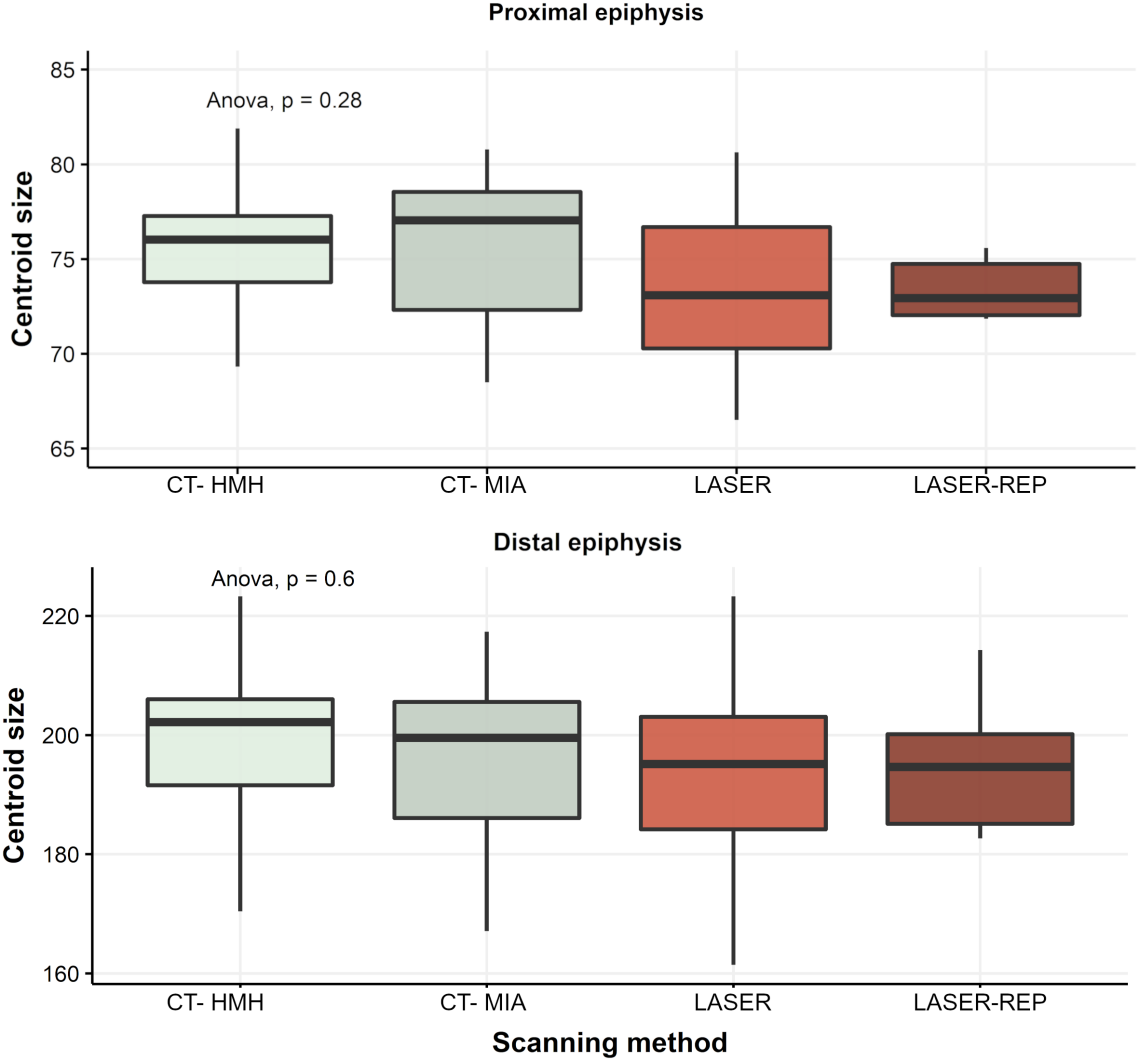
Centroid size comparisons among landmark configurations applied to computed tomography (CT)‐generated segmented with HMH, with MIA‐clustering (in gray), and laser scanner‐generated individuals and replicates (in red), displayed for both proximal and distal epiphysis and results of one‐way ANOVA.

Centroid size differences among CT‐generated meshes segmented with HMH and MIA‐clustering protocols and laser scanner replicates are appreciable but statistically not significant for both epiphyses (Figure 6). Overall, CT‐generated meshes either segmented with HMH and MIA‐clustering protocols tend to slightly overestimate mesh size, but ANOVA test confirm that for both epiphyses comparisons are not significant (*p* > 0.05).

## 4 DISCUSSION AND CONCLUSION

Both topological and geometric morphometric analysis demonstrated that CT scanning and laser scanning provide similar meshes of the fibula, fully comparable to one another, suggesting these acquisition devices could be adopted interchangeably in morphological analyses. Deviations among the meshes obtained by using the two devices are comparable with those reported in previous studies (Brzobohatá et al., 2012; Fahrni et al., 2017; Lalone et al., 2015; Waltenberger et al., 2021). Our results agree with those obtained on long bones by Soodmand et al. (2018), adopting a similar acquisition protocol: they found a mean root mean square error below 0.84 mm between CT and laser‐scanning meshes and reported consistent inter‐ observer agreement among processing of different laboratories acquiring and elaborating the same model. Our data are also consistent with those of Stephen et al. (2021), who examined a sample of long bones, which despite some differences in the CT scanning protocols in relation to slice thickness (1 mm instead of our 0.65 mm), found similar mean deviation values between CT and laser‐scanning meshes (tibia: 0.71–0.75 mm; femur: 0.69–0.80 mm), with no significant difference between surface accuracy and methodology. Our results on the fibula fall within this accuracy range, spanning an average of 0.35–0.56 mm when CT‐obtained meshes segmented with MIA‐clustering protocol are considered.

The comparison of our results to those of previous studies on comparability of surface reconstructions (Brzobohatá et al., 2012; Choi et al., 2002; DeVries et al., 2008; Fahrni et al., 2017; Ramme et al., 2009) should account for differences in protocols of acquiring and elaborating volumetric data, and the variety of the examined anatomic components (skull, phalanges). For instance, Brzobohatá et al. (2012), Choi et al. (2002), DeVries et al. (2008), Ramme et al. (2009) utilized a slice thickness of 1.0 mm, within their specific image acquisition parameters on medical CT scans. DeVries et al. (2008) revealed that the overall mean difference between the manually segmented CT models of hand phalanxes and their laser surface scan was 0.20 mm, with minimal effect of different smoothing procedures on surface representations, also coherent with our results (Table 1; Figure S1).

Concerning the smoothing procedures, our results highlight only minimal variation related to procedure choice (Table 2) in agreement with a previous work on smoothing procedures that have showed that smoothing algorithm affected mesh topology differently, despite retaining measurement accuracy in most reconstructions (Veneziano et al., 2018). While Veneziano et al. (2018) found that the Taubin algorithm ensured avoiding information loss on the surface of the mesh, we found a lower mean distance between the CT‐generated mesh smoothed with Laplacian algorithm with surface preserve. It is important to notice, though, that in our testing the best results were obtained when the iterations were minimal.

Regarding the segmentation protocols, our results indicate that different chosen single‐thresholding value influences topological differences of CT scan and laser scanner meshes, suggesting the necessity of a homogeneous segmentation protocol. Previous works (Engelbrecht et al., 2013; Fourie et al., 2012; Rathnayaka et al., 2011) found that, despite differences in accuracy of the 3D model in relation the segmentation methods, even the least accurate of the analyzed segmentation protocols (the single‐threshold technique) produced comparable 3D reconstruction, minimally deviating from their high‐resolution gold standard (0.18–0.24 mm). This is consistent with our study, where the adoption of a single‐threshold segmentation protocol provided average deviations mostly below the maximum resolution of the CT scan of 0.625 mm in slice thickness, even in cases of small cortical defects, despite with a less accurate performance. On the other hand, MIA‐clustering and HMH protocols offered non‐relevant deviations (−0.02–0.2 mm) at epiphyses and in larger portions of the diaphysis (Figure 3), compared to meshes generated with other segmentations based on single grayscale values, possibly reflecting shortcomings of single grayscale value thresholding in detecting thinner cortical feature on the bone surface. Indeed, Ito (2019) found that single grayscale value thresholding, including HMH protocol, might induce excessive erosion in 3D reconstructions, especially in low‐density areas, suggesting caution when combining segmentation protocols within a dataset for detailed GM analysis (e.g., asymmetry), since inter‐method error may be greater than shape variations explained by asymmetry. In fact, single thresholding segmentations may induce errors due to intensity inhomogeneity of CT data, and therefore may not capture fine bone surface features (Scherf & Tilgner, 2009). Adopting a segmentation protocol such as MIA‐clustering, based on a machine‐learning approach that adopts a global and local fuzzy c‐means clustering, here implemented on medical CT images, yielded better results than single‐thresholding segmentations, providing a practical, inexpensive solution to possible low resolution and grayscale intensity shortcomings (Dunmore et al., 2018). This segmentation approach could also be useful in clinical applications, for the 3D evaluation of the tibio‐fibular syndesmotic space, as it is a timesaving technique (Ebinger et al., 2013). Such variations may also have implications for GM analysis, as landmarks situated on sharp peaks may be sensitive to volume‐averaging effect, causing blurring in bone regions with low density or in adjacent bones with adjacent soft tissue (Wang et al., 2006).

In our results, we found that our landmark and semilandmark configurations mostly overlap in homologous individuals scanned with CT and laser scanner (Figure 5) and only 1.38%–1.43% of total variance in the dataset is explained by difference in scanning device, with no statistically significant differences in morphology between the two methods. The results of our PCA analyses (Figure 5) are comparable to those of Marcy et al. (2018) and Waltenberger et al. (2021), even when both sets of scanning digitization are added, with each individual, either CT‐ or laser scanned, clustering together. However, while Waltenberger et al. (2021) found no significant difference in their Procrustes ANOVA in accordance with our own results, Marcy et al. (2018), who compared laser scanner and micro‐CT derived mesh reconstructions of mice skulls, found that the percentage of variance explained by asymmetry (directional and fluctuating) was lower than the percentage of variance explained by scanning device. According to the authors, this would suggest that analyses of asymmetry with a combination of scanning methods scans may be subject to systematic error. In both ours and Waltenberger et al. (2021) cases, it is possible that the relatively big size of the fibula in contrast to the diminutive size of mice skulls may have prevented this effect, therefore supporting the comparability of scanning methodology through geometric morphometric methods and the combination of different datasets in GM analysis of human bone elements. Moreover, as in our sample 98.57%–98.62% of variance is explained by factors other than scanning methodology, it is possible to infer that such shape variations may account for biological diversity within our sample.

Centroid size comparisons for both epiphyses revealed that, while CT‐generated meshes are larger than laser scanner‐generated meshes, their size is comparable. The larger centroid size of CT‐generated meshes also confirms the observations of Table 3, with mean distances between CT‐generated meshes and laser scanner‐generated meshes indicating all positive values, suggesting that the latter meshes possess indeed smaller surface and areas than the formers.

In our analysis, inter‐method comparisons showed good reproducibility of landmark digitization, with lower Procrustes distance between each set of CT and laser scanner replicates than inter‐methods, suggesting low intra‐observer error. This is consistent with Fruciano et al. (2017), who found that when combining landmark data from multiple devices and digitized by multiple operators and test for the presence of bias, a larger amount of variance was explained by the operator compared to the device. The authors however also found that, similar to Robinson and Terhune (2017), inter‐method and inter‐observer errors may be greater than small intraspecific or closely related species shape variations. Indeed, the authors advocate for testing accuracy trials of different methods and comparability of observers prior to combination of different data source.

In conclusion, we validated the comparability and combination of 3D reconstruction of human fibulae, generated by dual‐energy CT scan and two sets of 3D laser scanner replicates, confirming substantial topological similarity of the meshes reconstructed with the two methods and corroborating their interchangeability in GM studies, as no significant inter‐method and intra‐observer difference is found in shape analysis, with potential applications in clinical context (Souleiman et al., 2021). A possible limitation of the present study is the use of laser scanner‐generated meshes as the gold standard comparison from which distances of CT‐generated meshes are calculated, assuming their closeness to the real bone surface as being more accurate. In our analyses, in fact, we did not evaluate this proximity to the real specimens, but on the basis of previous evaluations utilizing the same laser scanner employed here (Adams et al., 2015), and given that the 3D accuracy stated by the manufacturer is to 50 μm, we could indeed infer that the laser scanner surfaces are the most accurate approximations. We suggest, in accordance with previous studies (Marcy et al., 2018; Robinson & Terhune, 2017; Waltenberger et al., 2021), that prior to formal analysis, coherence of both CT and laser scanning protocols, including assessment of scanning settings and resolutions, segmentations and reconstruction post‐processing, should be thoroughly evaluated, in case of merging different data sources, possibly with dedicated trials.

## AUTHORS' CONTRIBUTIONS

AP conceptualization, investigation, formal analysis, validation, writing‐original draft, methodology, writing‐review, and editing; RS validation, writing‐original draft, methodology, writing‐review, and editing; VN data acquisition, formal analysis, validation; SD conceptualization, supervision of the research, methodology, data acquisition, validation, software; DM, SB, MGB conceptualization, supervision of the research, methodology, project administration, software, writing‐review and editing.

## CONFLICT OF INTEREST

The authors declare no potential conflict of interest.

## Supporting information


Figure S1
Figure S2
Figure S3
Figure S4
Figure S5
Figure S6
Figure S7
Figure S8
Figure S9
Figure S10
Figure S11
Figure S12
Figure S13
Figure S14
Table S1
Appendix S1
Appendix S2

## Data Availability

All data acquired and analyzed in this work is available upon request addressed to the corresponding author. Landmarks coordinates and sample lists for geometric morphometric analysis are available (DOI: 10.5281/zenodo.6425379).
