# Supplementary material for: Comparability of skeletal fibulae surfaces generated by different source scanning (dual‐energy CT scan vs. high resolution laser scanning) and 3D geometric morphometric validation
Source: J Anat. 2022 Jun 25;241(3):667–82. doi: 10.1111/joa.13714 (PMC9358749; doi:10.1111/joa.13714)
Supplement: Supplementary file 1 — Figure S1 Figure S2 Figure S3 Figure S4 Figure S5 Figure S6 Figure S7 Figure S8 Figure S9 Figure S10 Figure S11 Figure S12 Figure S13 Figure S14 Table S1 Appendix S1 Appendix S2 [file JOA-241-667-s001.docx]

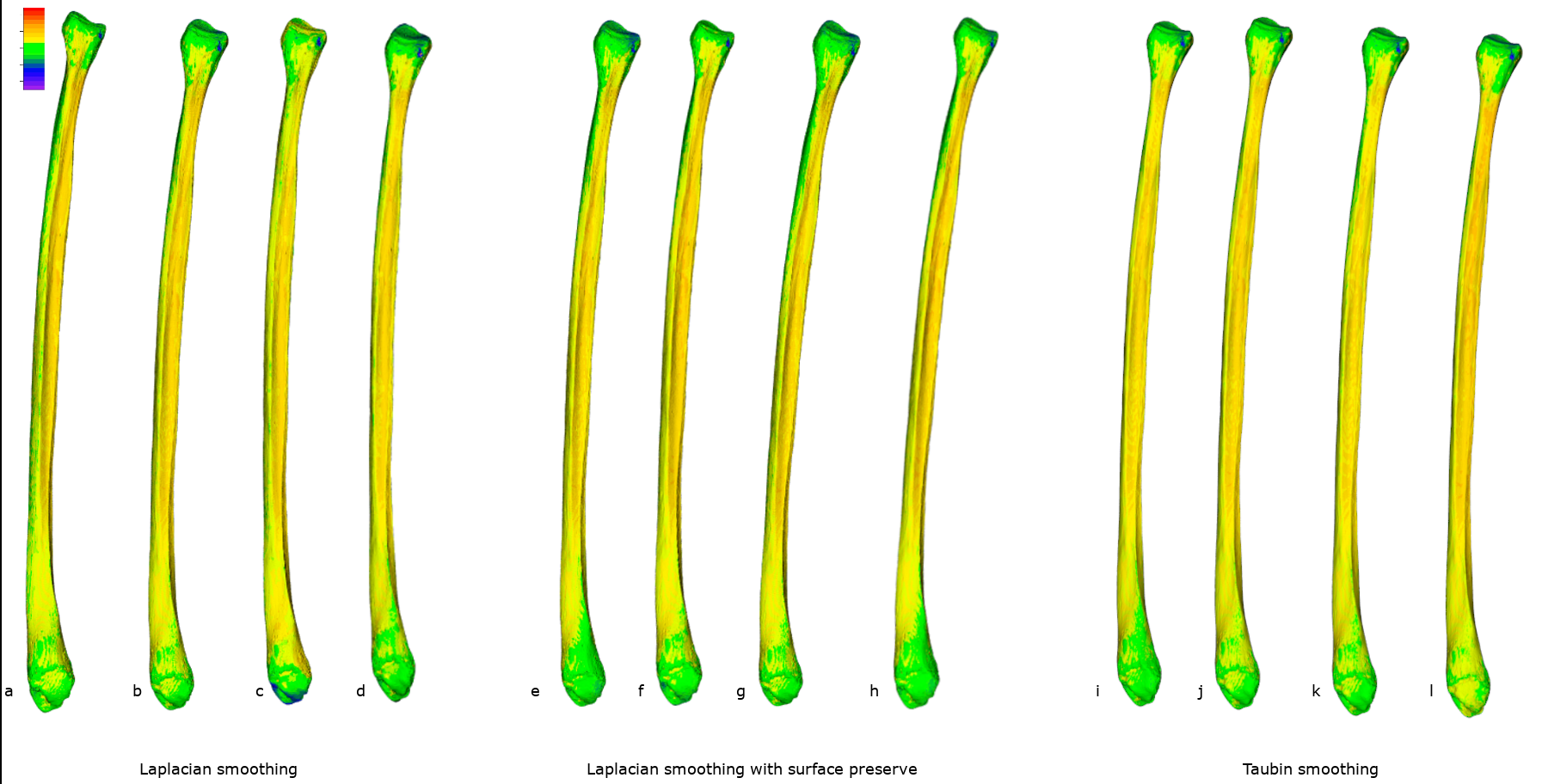


**Figure S1.** Colormaps (-2.5 to 2.5 mm, from violet to red; in white, deviations exceed this range) of vertex distances calculated comparing CT-generated mesh of SS163, segmented with HMH protocol, and subjected to different smoothing protocols, and its respective laser scanner- generated mesh. From left to right, Laplacian smoothing at 0.5 (a), 1 (b), 3 (c) and 5 (d) iterations; Laplacian smoothing with surface preserve at 0.5 (e), 1 (f), 3 (g) and 5 (h) iterations; Taubin smoothing at 0.5 (i), 1 (j), 3 (k) and 5 (l) iterations.

|  | **Maximum diameter at midshaft (mm)** |
| --- | --- |
| **SS115** | 15.85 |
| **SS163** | 14.31 |
| **SS224** | 15.73 |
| **SS234** | 14.56 |
| **SS28** | 11.12 |
| **SS26** | 16.88 |
| **SS234** | 16.35 |
| **SS48** | 14.38 |
| **SS105** | 17.64 |
| **PAN** | 12.62 |
| **SS4** | 17.17 |
| **SS14** | 14.19 |
| **SS215** | 13.83 |
| **SS95** | 13.90 |
| **SS115** | 15.98 |

**Table S1.** Maximum diameter calculated at fibular midshaft for each specimen. Values are in mm.


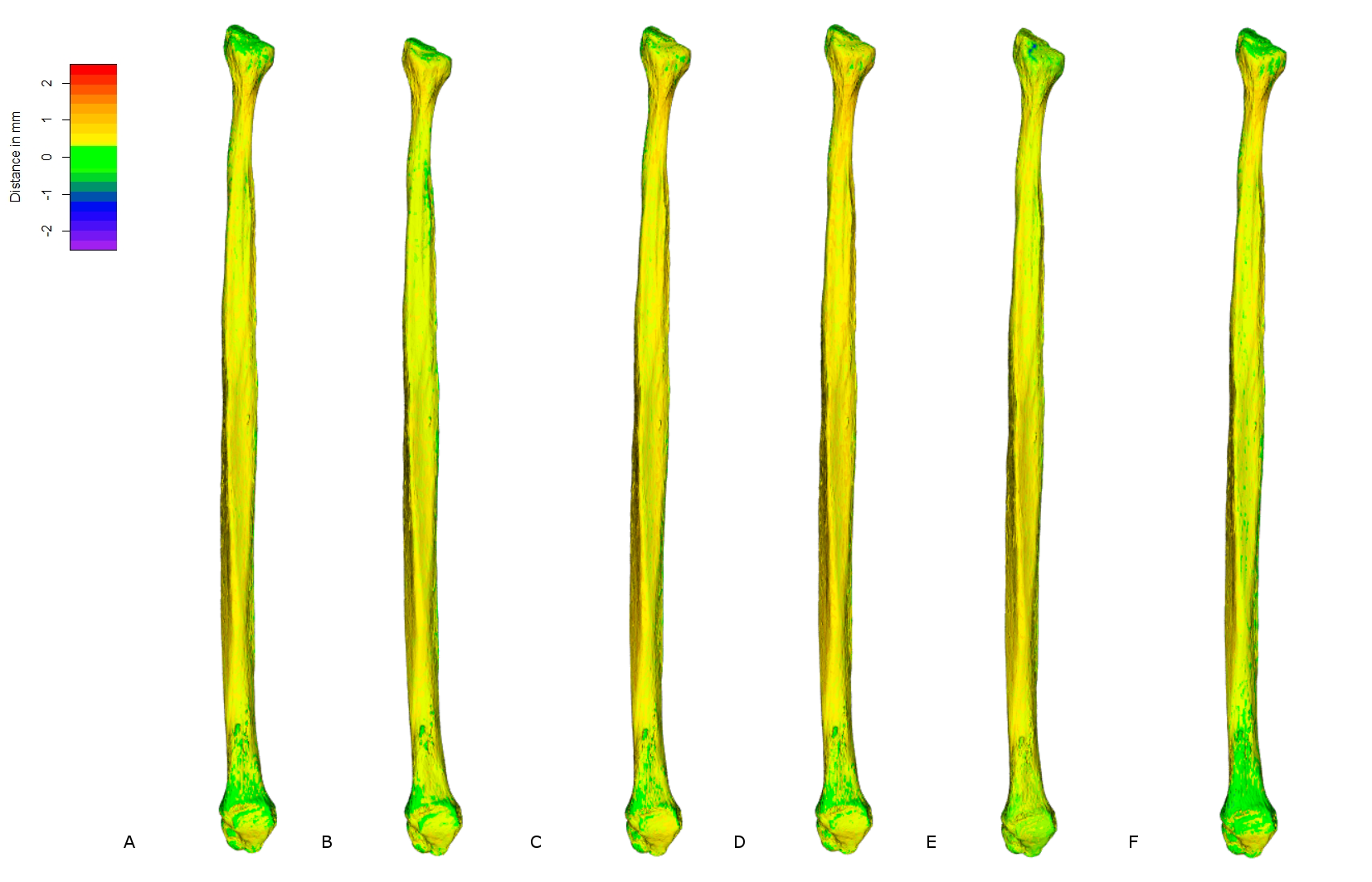


**Figure S2**. Computed tomography (CT) -generated meshes for every segmentation protocol SS4, with relative distances (-2.5 to 2.5 mm, from violet to red; in white, areas where deviations exceed this range), from their laser-scanner generated equivalent. a, CT segmented at -550 grayscale intensity; b, CT segmented at -600 grayscale intensity; c, CT segmented at -650 grayscale intensity ; d, CT segmented at -700 grayscale intensity ; and e, CT segmented with Half Maximum Height protocol (HMH) and f, MIA-clustering protocol (MIA).


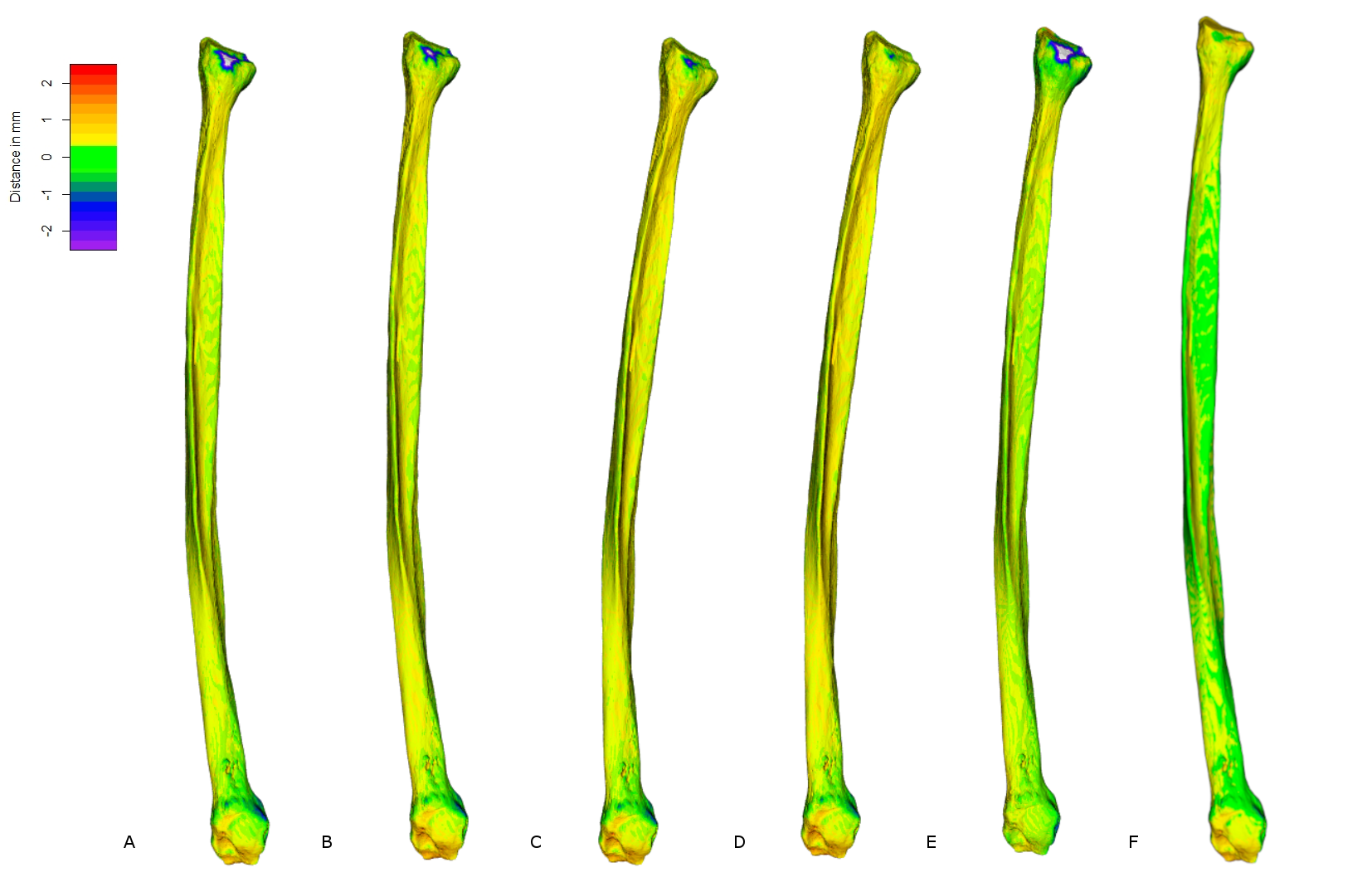


**Figure S3**. Computed tomography (CT) -generated meshes for every segmentation protocol for SS14, with relative distances (-2.5 to 2.5 mm, from violet to red; in white, areas where deviations exceed this range), from their laser-scanner generated equivalent. a, CT segmented at -550 grayscale intensity; b, CT segmented at -600 grayscale intensity; c, CT segmented at -650 grayscale intensity ; d, CT segmented at -700 grayscale intensity ; and e, CT segmented with Half Maximum Height protocol (HMH) and f, MIA-clustering protocol (MIA).


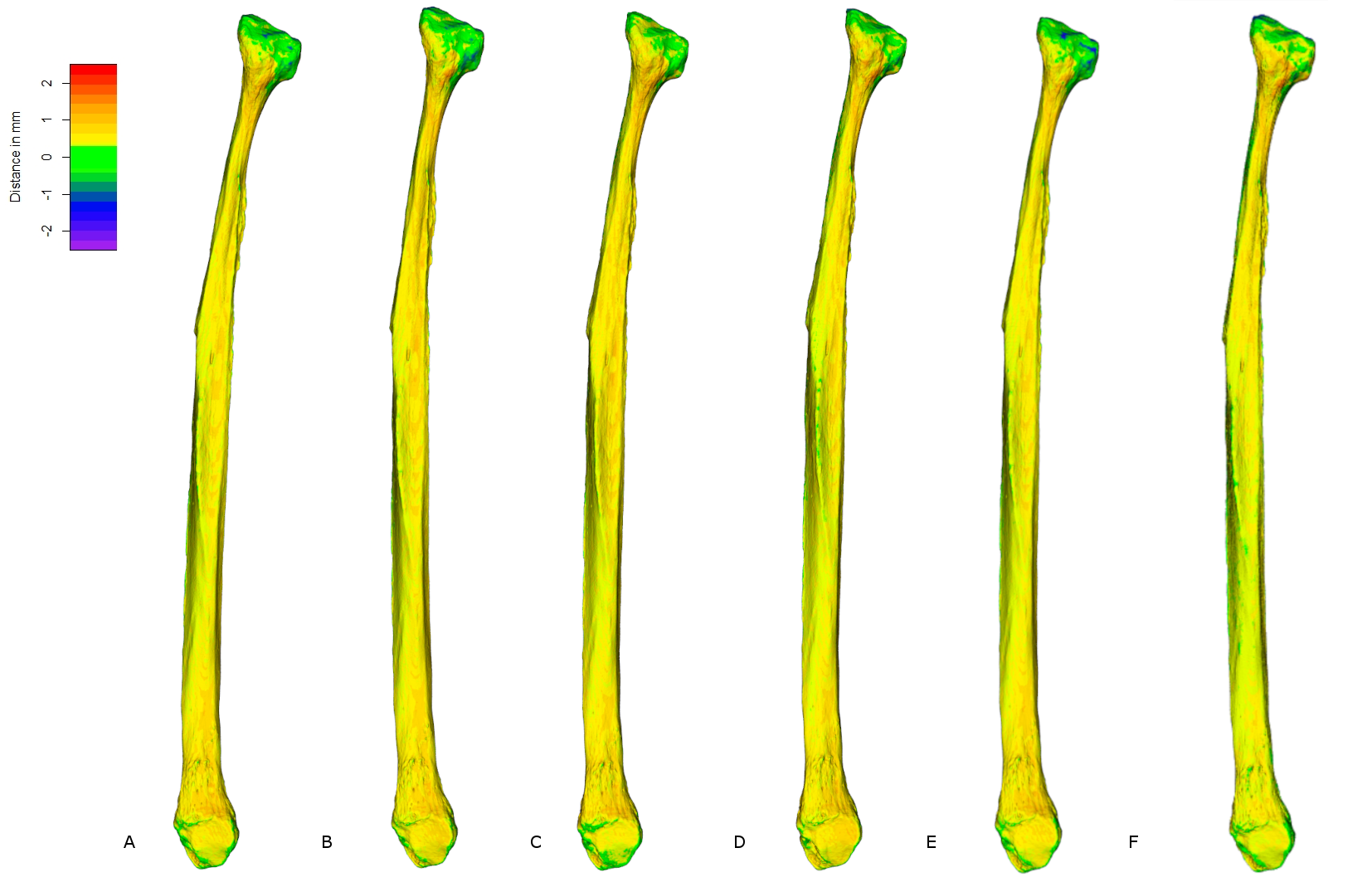


**Figure S4**. Computed tomography (CT) -generated meshes for every segmentation protocol for SS26, with relative distances (-2.5 to 2.5 mm, from violet to red; in white, areas where deviations exceed this range), from their laser-scanner generated equivalent. a, CT segmented at -550 grayscale intensity; b, CT segmented at -600 grayscale intensity; c, CT segmented at -650 grayscale intensity ; d, CT segmented at -700 grayscale intensity ; and e, CT segmented with Half Maximum Height protocol (HMH) and f, MIA-clustering protocol (MIA).


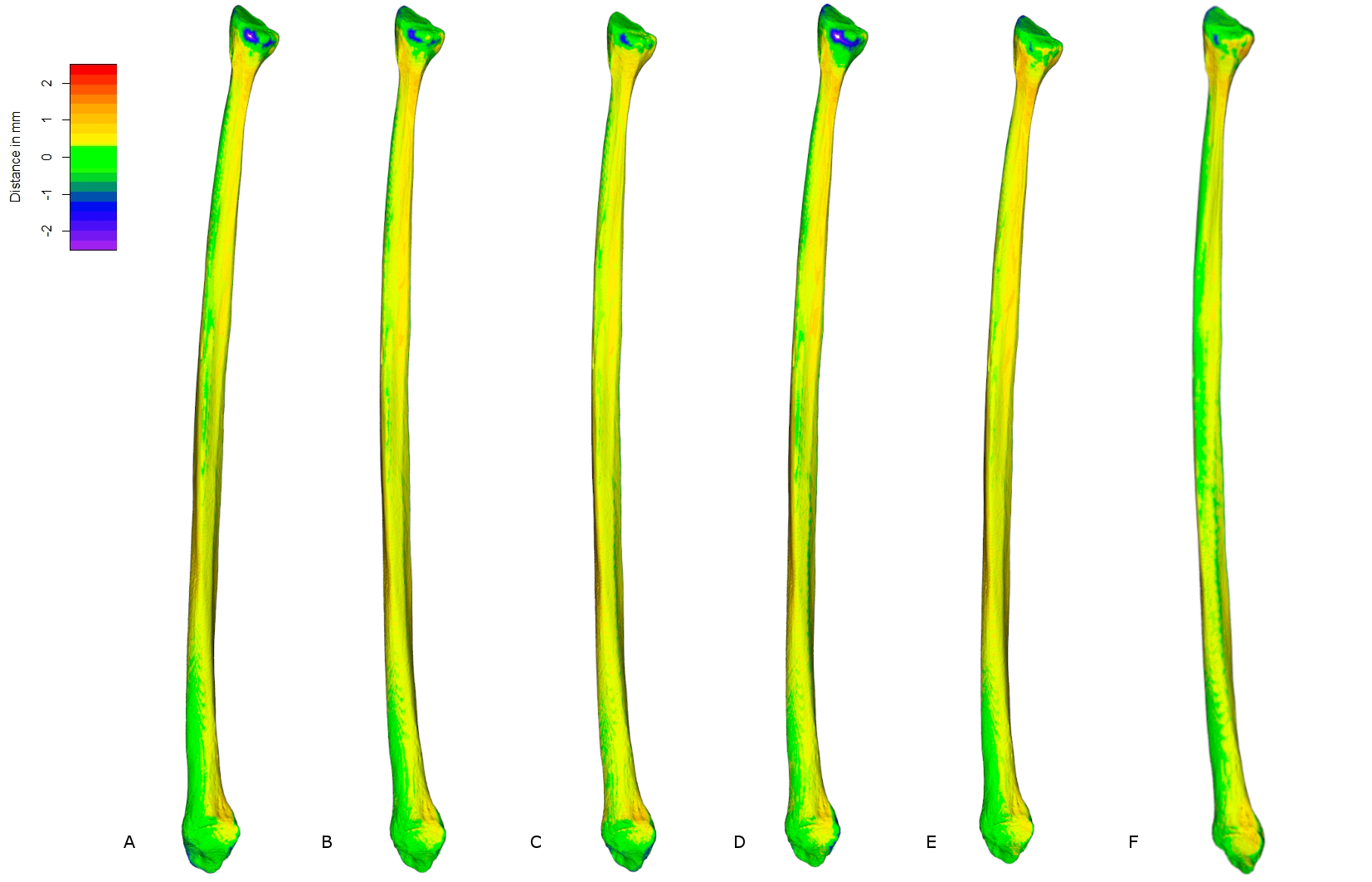


**Figure S5**. Computed tomography (CT) -generated meshes for every segmentation protocol for SS28, with relative distances (-2.5 to 2.5 mm, from violet to red; in white, areas where deviations exceed this range), from their laser-scanner generated equivalent. a, CT segmented at -550 grayscale intensity; b, CT segmented at -600 grayscale intensity; c, CT segmented at -650 grayscale intensity ; d, CT segmented at -700 grayscale intensity ; and e, CT segmented with Half Maximum Height protocol (HMH) and f, MIA-clustering protocol (MIA).


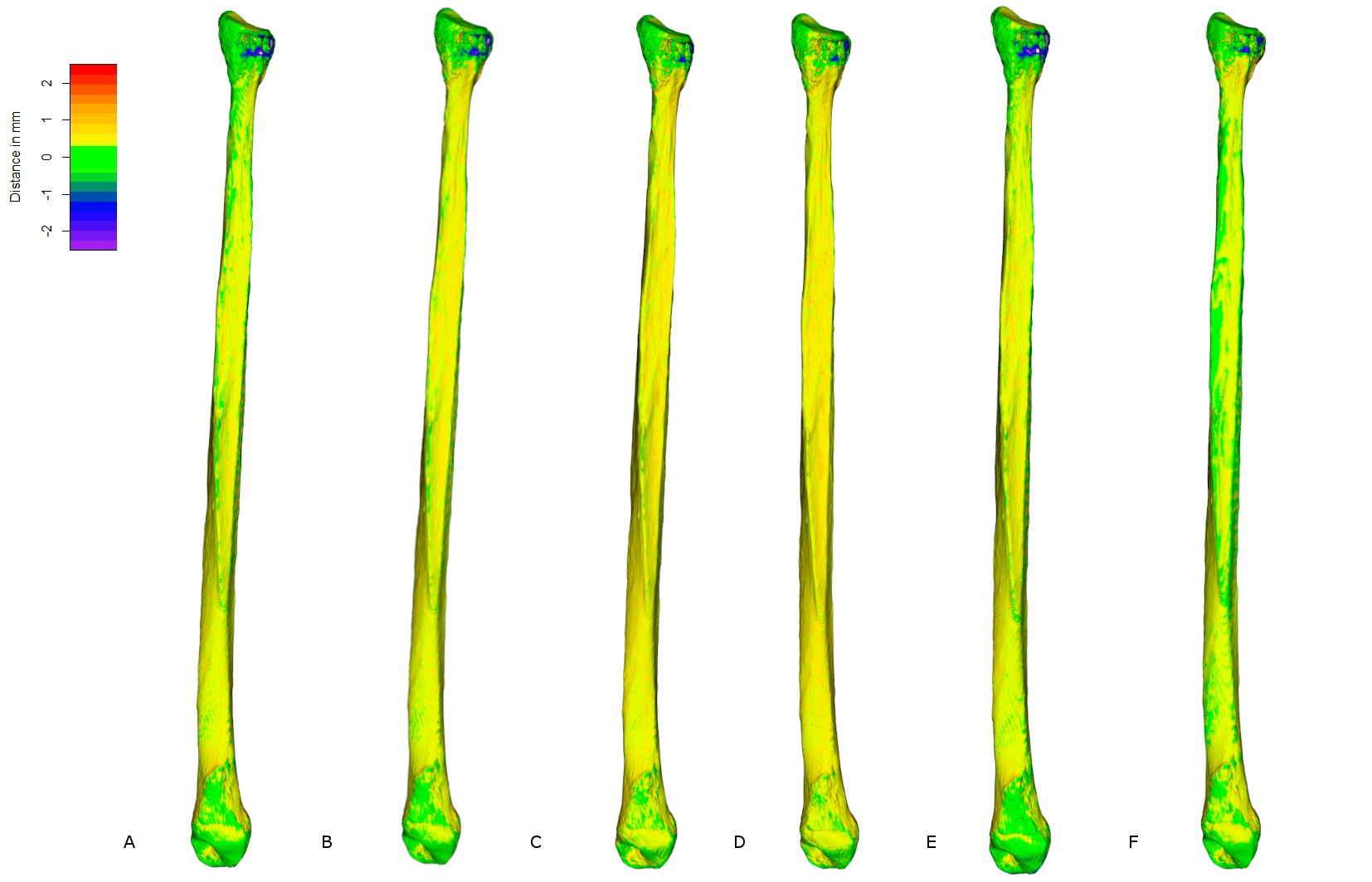


**Figure S6**. Computed tomography (CT) -generated meshes for every segmentation protocol for SS48, with relative distances (-2.5 to 2.5 mm, from violet to red; in white, areas where deviations exceed this range), from their laser-scanner generated equivalent. a, CT segmented at -550 grayscale intensity; b, CT segmented at -600 grayscale intensity; c, CT segmented at -650 grayscale intensity ; d, CT segmented at -700 grayscale intensity ; and e, CT segmented with Half Maximum Height protocol (HMH) and f, MIA-clustering protocol (MIA).


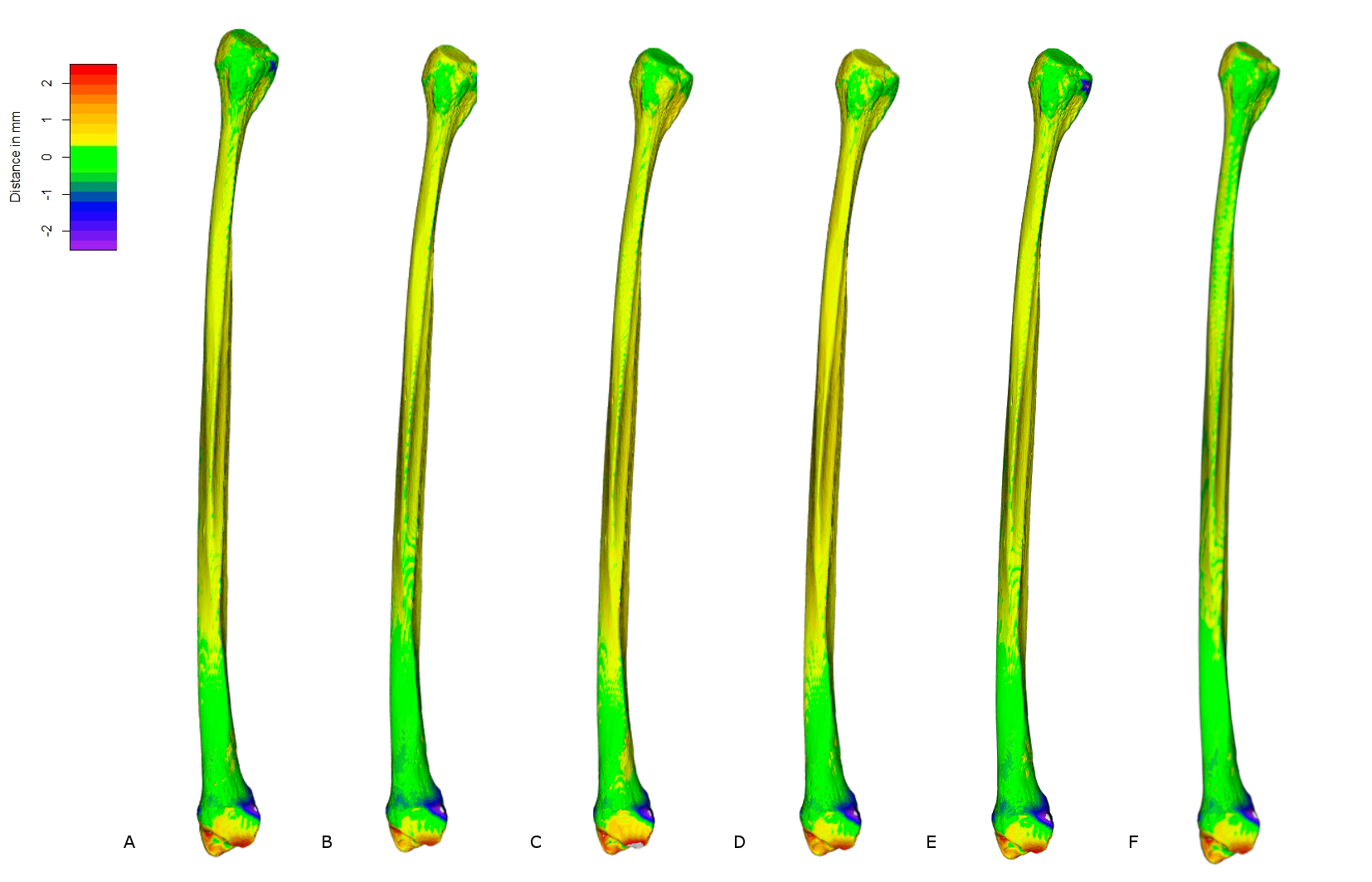


**Figure S7**. Computed tomography (CT) -generated meshes for every segmentation protocol for SS95, with relative distances (-2.5 to 2.5 mm, from violet to red; in white, areas where deviations exceed this range), from their laser-scanner generated equivalent. a, CT segmented at -550 grayscale intensity; b, CT segmented at -600 grayscale intensity; c, CT segmented at -650 grayscale intensity ; d, CT segmented at -700 grayscale intensity ; and e, CT segmented with Half Maximum Height protocol (HMH) and f, MIA-clustering protocol (MIA).


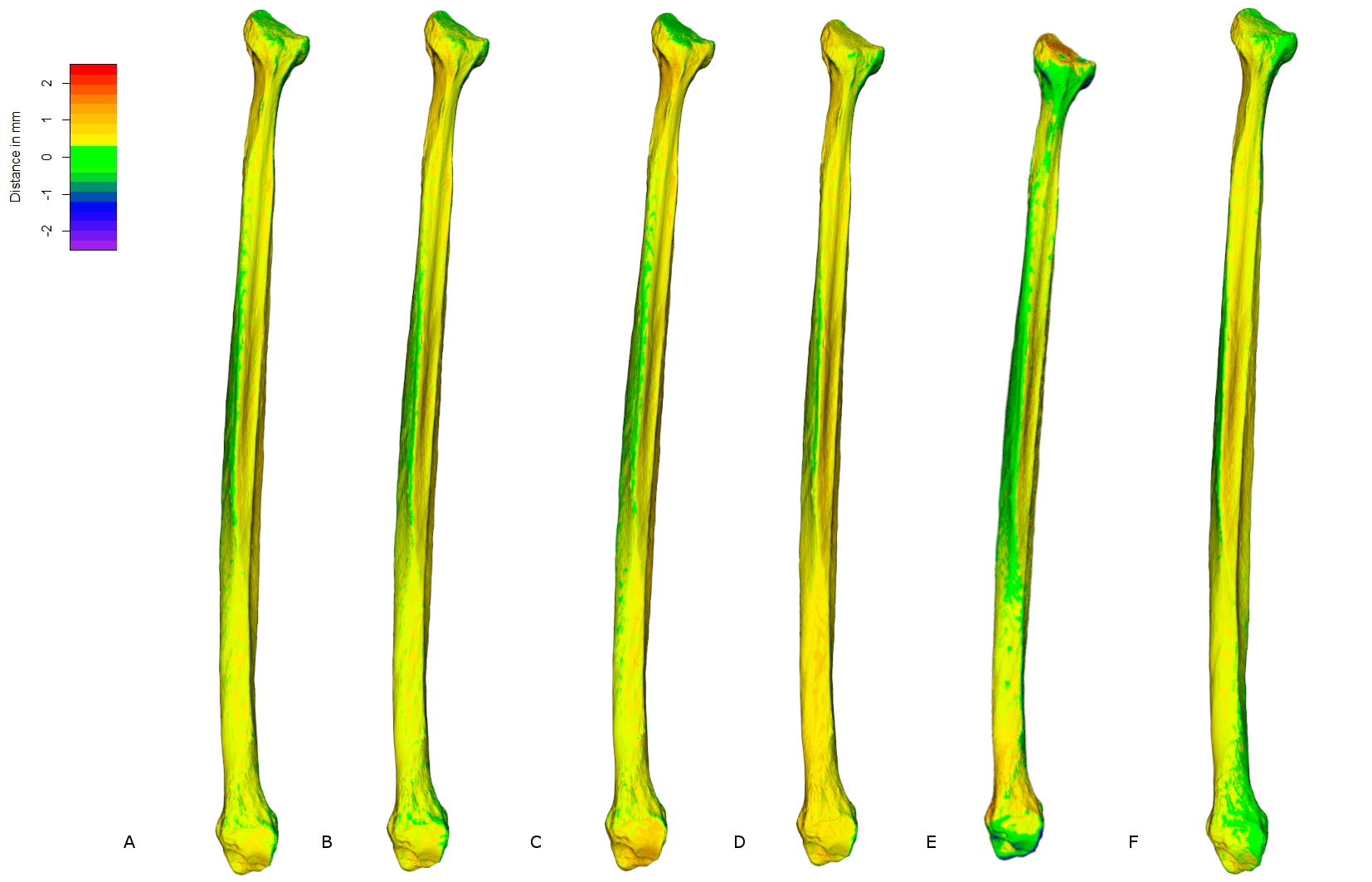


**Figure S8**. Computed tomography (CT) -generated meshes for every segmentation protocol for SS105, with relative distances (-2.5 to 2.5 mm, from violet to red; in white, areas where deviations exceed this range), from their laser-scanner generated equivalent. a, CT segmented at -550 grayscale intensity; b, CT segmented at -600 grayscale intensity; c, CT segmented at -650 grayscale intensity ; d, CT segmented at -700 grayscale intensity ; and e, CT segmented with Half Maximum Height protocol (HMH) and f, MIA-clustering protocol (MIA).


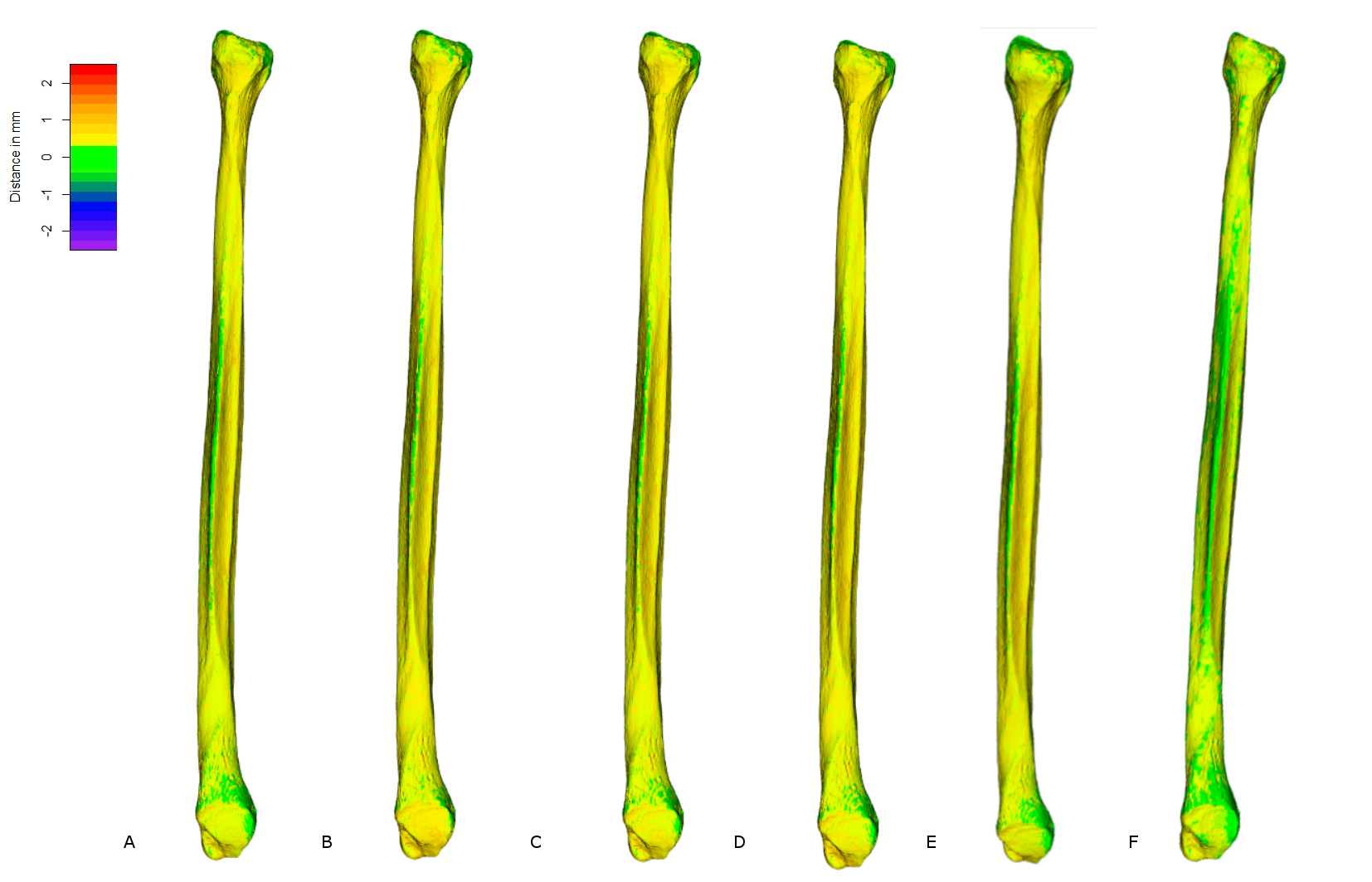


**Figure S9**. Computed tomography (CT) -generated meshes for every segmentation protocol for SS115, with relative distances (-2.5 to 2.5 mm, from violet to red; in white, areas where deviations exceed this range), from their laser-scanner generated equivalent. a, CT segmented at -550 grayscale intensity; b, CT segmented at -600 grayscale intensity; c, CT segmented at -650 grayscale intensity ; d, CT segmented at -700 grayscale intensity ; and e, CT segmented with Half Maximum Height protocol (HMH) and f, MIA-clustering protocol (MIA).


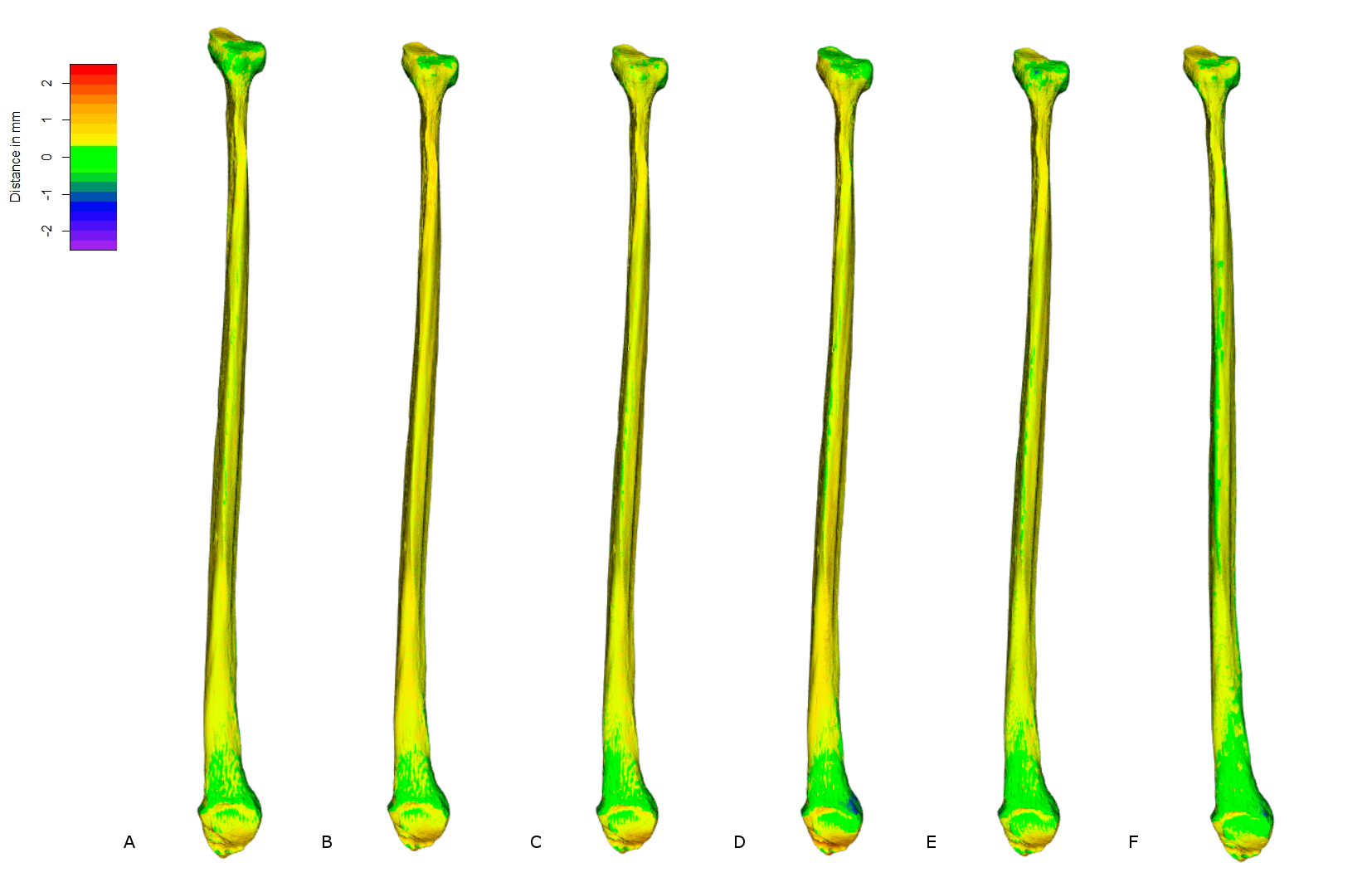


**Figure S10**. Computed tomography (CT) -generated meshes for every segmentation protocol for SS215, with relative distances (-2.5 to 2.5 mm, from violet to red; in white, areas where deviations exceed this range), from their laser-scanner generated equivalent. a, CT segmented at -550 grayscale intensity; b, CT segmented at -600 grayscale intensity; c, CT segmented at -650 grayscale intensity ; d, CT segmented at -700 grayscale intensity ; and e, CT segmented with Half Maximum Height protocol (HMH) and f, MIA-clustering protocol (MIA).


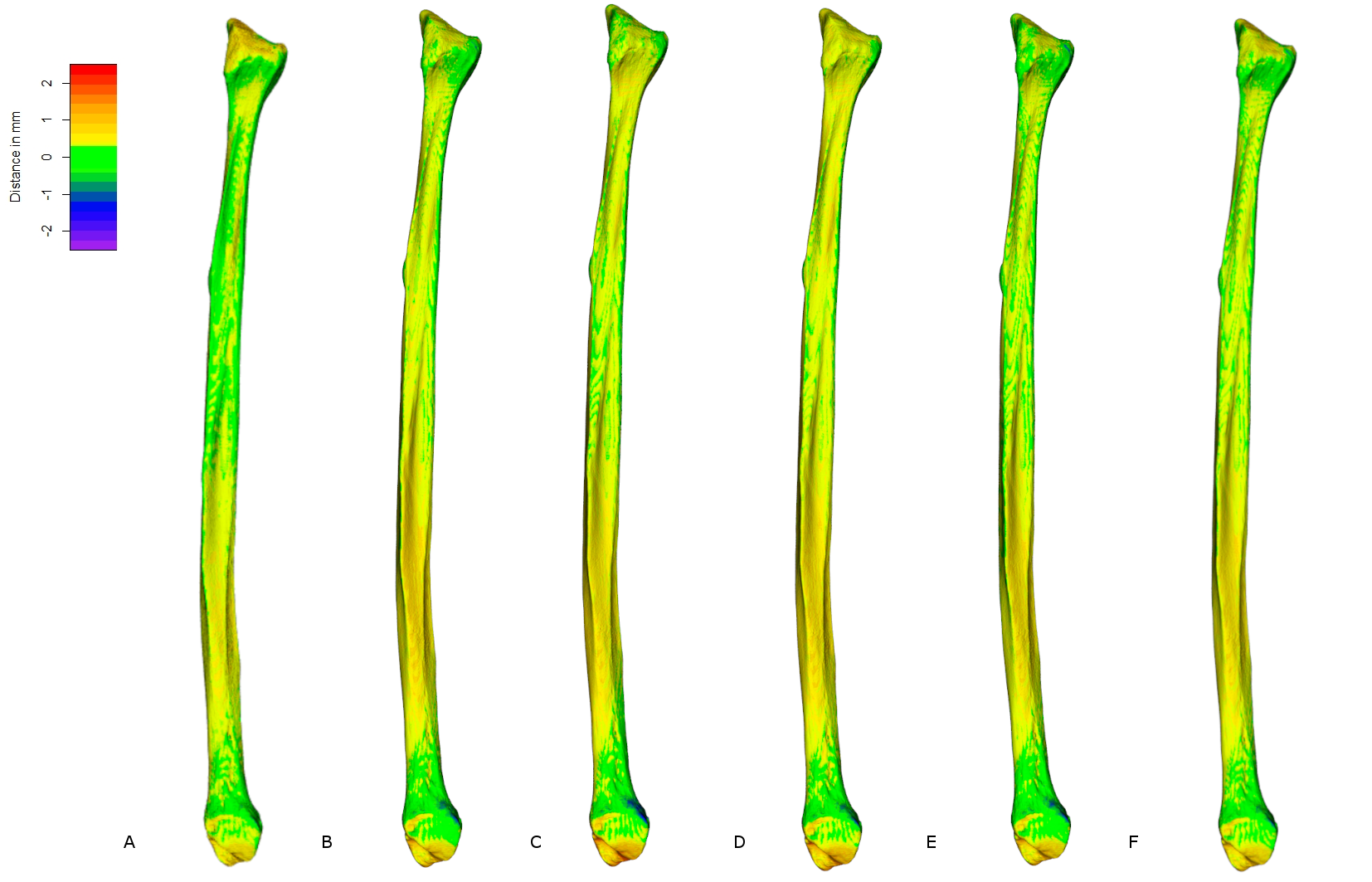


**Figure S11**. Computed tomography (CT) -generated meshes for every segmentation protocol for SS224, with relative distances (-2.5 to 2.5 mm, from violet to red; in white, areas where deviations exceed this range), from their laser-scanner generated equivalent. a, CT segmented at -550 grayscale intensity; b, CT segmented at -600 grayscale intensity; c, CT segmented at -650 grayscale intensity ; d, CT segmented at -700 grayscale intensity ; and e, CT segmented with Half Maximum Height protocol (HMH) and f, MIA-clustering protocol (MIA).


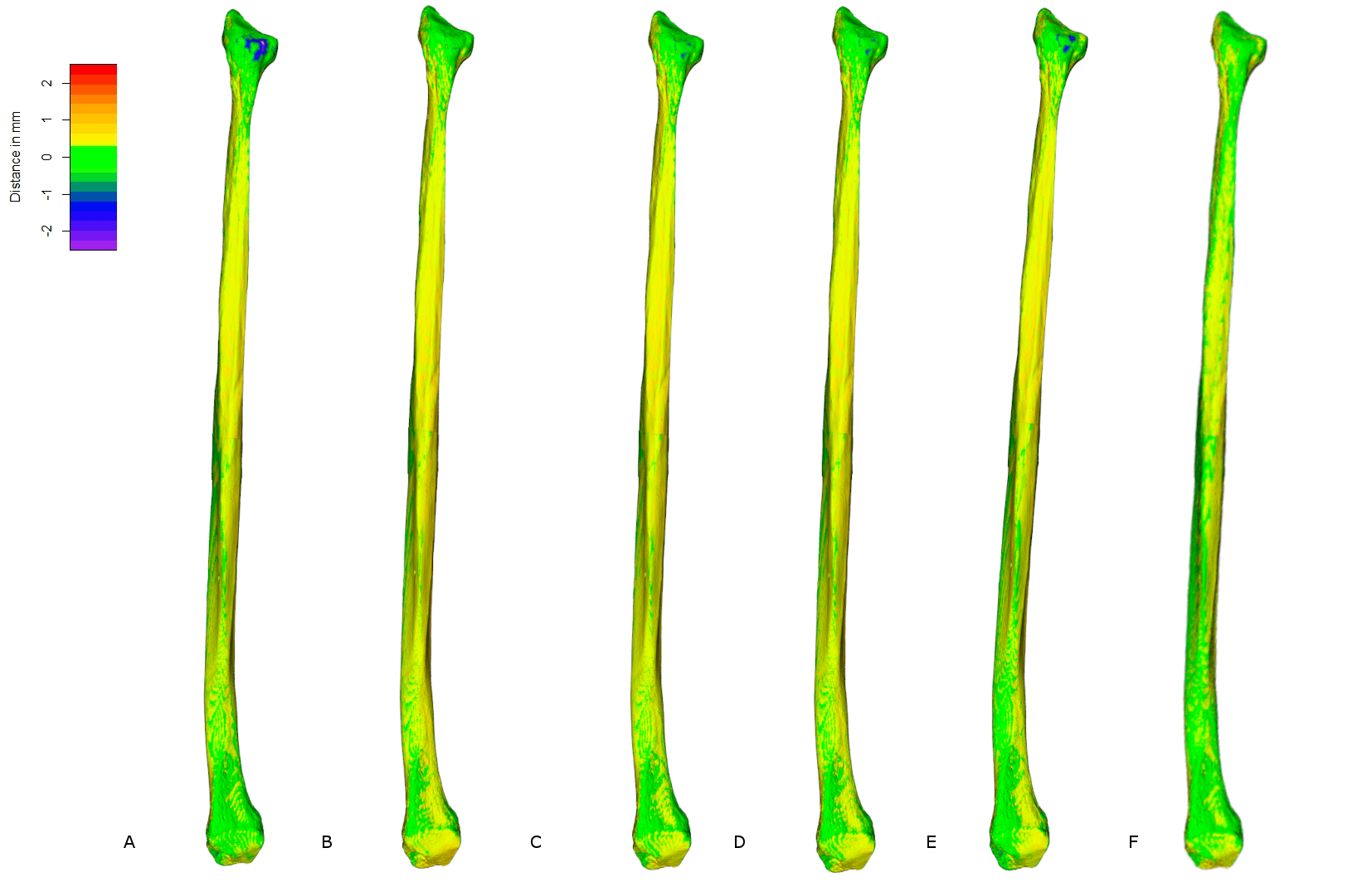


**Figure S12**. Computed tomography (CT) -generated meshes for every segmentation protocol for SS234, with relative distances (-2.5 to 2.5 mm, from violet to red; in white, areas where deviations exceed this range), from their laser-scanner generated equivalent. a, CT segmented at -550 grayscale intensity; b, CT segmented at -600 grayscale intensity; c, CT segmented at -650 grayscale intensity ; d, CT segmented at -700 grayscale intensity ; and e, CT segmented with Half Maximum Height protocol (HMH) and f, MIA-clustering protocol (MIA).


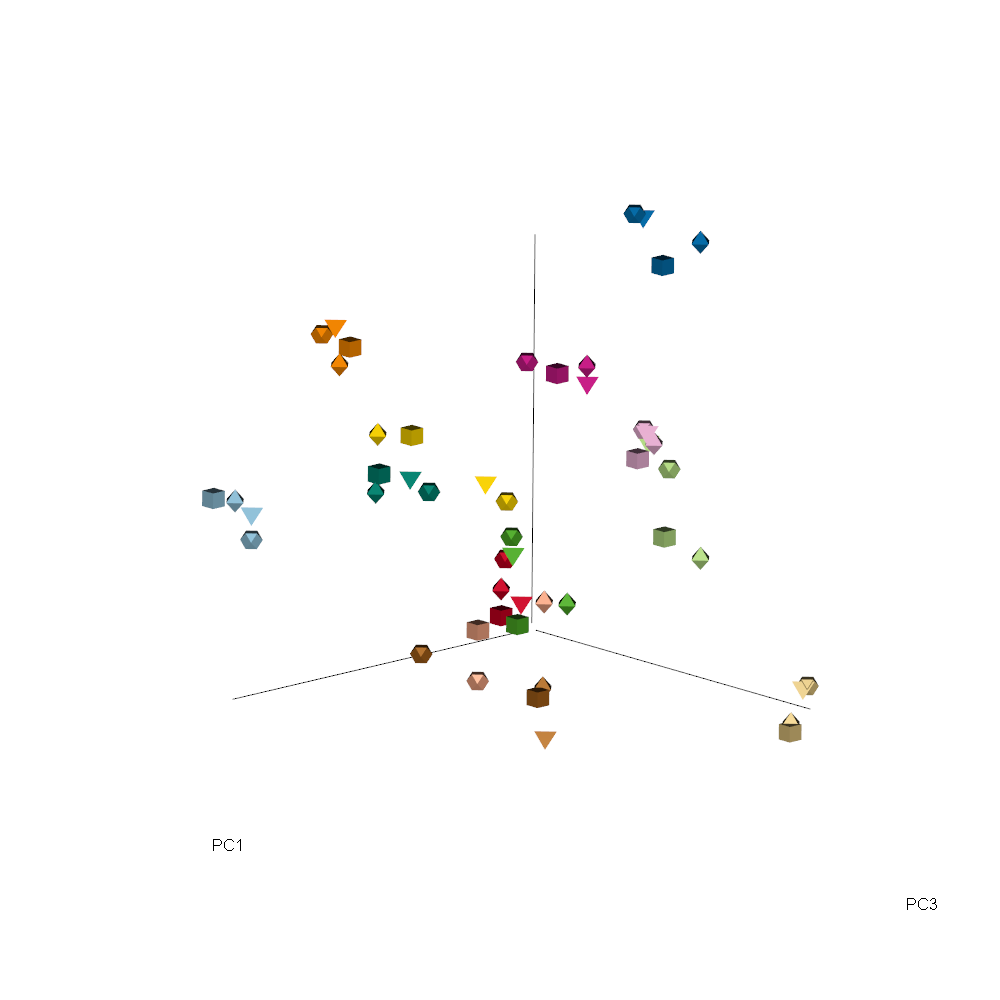


**Figure S13.** 3D Principal component analysis plot considering landmark configurations of proximal epiphysis applied to computed tomography (CT)-generated meshes segmented with HMH (cubes) protocol and MIA-clustering protocol (octahedron) and laser scanner-generated specimens (original= tetrahedron; replica= cuboctahedron).


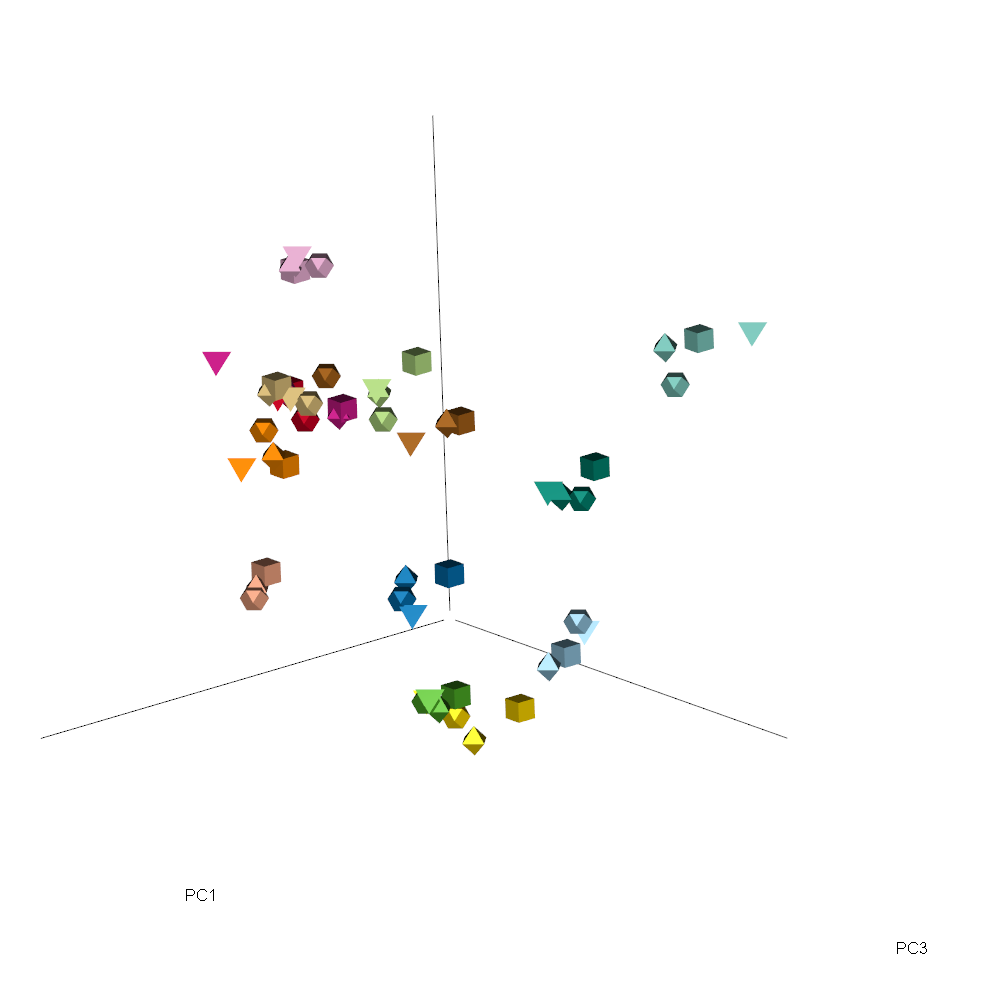


**Figure S14.** 3D Principal component analysis plot considering landmark configurations of distal epiphysis applied to computed tomography (CT)-generated meshes segmented with HMH (cubes) protocol and MIA-clustering protocol (octahedron) and laser scanner-generated specimens (original= tetrahedron; replica= cuboctahedron).

**SI_Appendix 1.** Matrix of Cartesian coordinates of analyzed specimens (DOI: 10.5281/zenodo.6425379).

**SI_Appendix 2.** Sample list and acquisition methods (DOI: 10.5281/zenodo.6425379).
